# Supplementary material for: Trajectory tracking sliding mode control for vertical take-off and landing aircraft based on double loop and global Lipschitz stability
Source: PLoS One. 2025 Feb 7;20(2):e0318294. doi: 10.1371/journal.pone.0318294 (PMC11805409; doi:10.1371/journal.pone.0318294)
Supplement: S1 File — (PDF) [file pone.0318294.s001.pdf]

%%%%%%%%%%%%%% Main Code

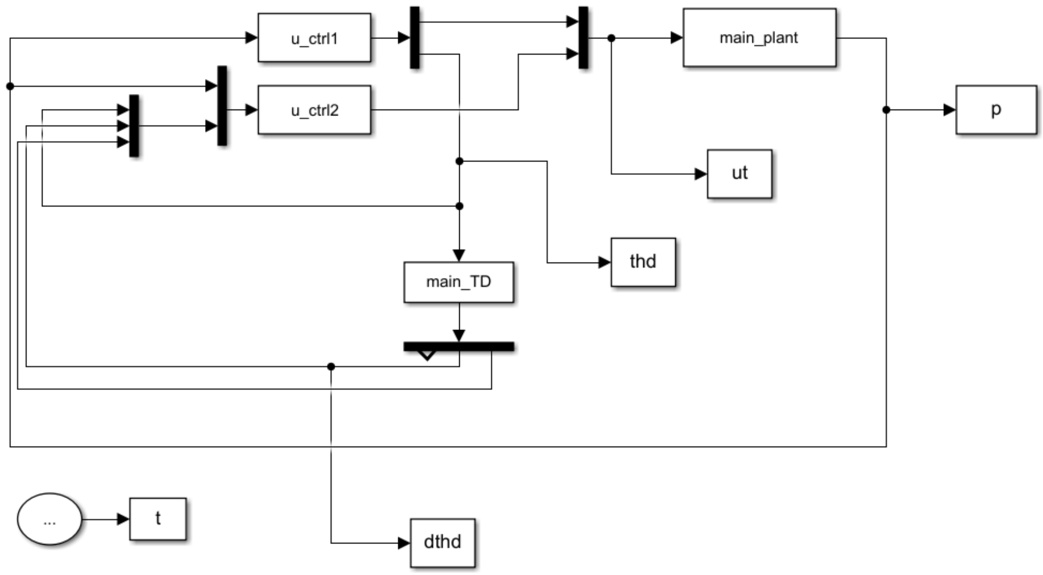

%%%%%%%%%%%%%% Simulink Model

%%%%%%%%%%%%% main_plant

```
function [sys,x0,str,ts]=s_function(t,x,u,flag)
switch flag,
case 0,
    [sys,x0,str,ts]=mdlInitializeSizes;
case 1,
    sys=mdlDerivatives(t,x,u);
case 3,
    sys=mdlOutputs(t,x,u);
case {2, 4, 9 }
    sys = [];
otherwise
    error(['Unhandled flag = ',num2str(flag)]);
end
function [sys,x0,str,ts]=mdlInitializeSizes
```

```
sizes = simsizes;
sizes.NumContStates    = 6;
sizes.NumDiscStates    = 0;
sizes.NumOutputs          = 6;
sizes.NumInputs           =2;
sizes.DirFeedthrough = 0;
sizes.NumSampleTimes = 1;
sys=simsizes(sizes);
x0=[0 0 0 0 0 0];
str=[];
ts=[-1 0];
function sys=mdlDerivatives(t,x,u)
th=x(5);
epc=0.88;g=9.8;

sys(1)=x(2);
sys(2)=-u(1)*sin(th)+epc*u(2)*cos(th);
sys(3)=x(4);
sys(4)=u(1)*cos(th)+epc*u(2)*sin(th)-g;
sys(5)=x(6);
sys(6)=u(2);
function sys=mdlOutputs(t,x,u)
x1=x(1);x2=x(2);
y1=x(3);y2=x(4);
th=x(5);dth=x(6);
sys(1)=x1;
sys(2)=x2;
```

```
sys(3)=y1;
sys(4)=y2;
sys(5)=th;
sys(6)=dth;

%%%%%%%%%%%%%%%%%%%%%%%%%%%%%%%    main_TD
function [sys,x0,str,ts] = spacemodel(t,x,u,flag)
switch flag,
case 0,
    [sys,x0,str,ts]=mdlInitializeSizes;
case 1,
    sys=mdlDerivatives(t,x,u);
case 3,
    sys=mdlOutputs(t,x,u);
case {2,4,9}
    sys=[];
otherwise
    error(['Unhandled flag = ',num2str(flag)]);
end
function [sys,x0,str,ts]=mdlInitializeSizes
sizes = simsizes;
sizes.NumContStates   = 3;
sizes.NumDiscStates   = 0;
sizes.NumOutputs      = 3;
sizes.NumInputs       = 1;
sizes.DirFeedthrough = 1;
sizes.NumSampleTimes = 1;
```

```
sys = simsizes(sizes);
x0    = [0 0 0];
str = [];
ts    = [0 0];
function sys=mdlDerivatives(t,x,u)
v=u(1);
a1=9;b1=27;c1=27;
kexi=0.01;
  if t<=1
          kexi=1/(100*(1-exp(-2*t)));
  end
sys(1)=x(2);
sys(2)=x(3);
sys(3)=-a1*(x(1)-v)/kexi^3-b1*x(2)/kexi^2-c1*x(3)/kexi;
function sys=mdlOutputs(t,x,u)
v=u(1);
sys(1)=v;
sys(2)=x(2);
sys(3)=x(3);


%%%%%%%%%%%%%%%%%%%%%     u_ctrl1
function [sys,x0,str,ts]=s_function(t,x,u,flag)
switch flag,
case 0,
        [sys,x0,str,ts]=mdlInitializeSizes;
case 3,
        sys=mdlOutputs(t,x,u);
```

```
case {1, 2, 4, 9 }
        sys = [];
otherwise
        error(['Unhandled flag = ',num2str(flag)]);
end
function [sys,x0,str,ts]=mdlInitializeSizes
sizes = simsizes;
sizes.NumDiscStates    = 0;
sizes.NumOutputs         = 2;
sizes.NumInputs          = 6;
sizes.DirFeedthrough = 1;
sizes.NumSampleTimes = 1;
sys=simsizes(sizes);
x0=[];
str=[];
ts=[0 0];
function sys=mdlOutputs(t,x,u)
g=9.8;epc=0.88;

x1=u(1);x2=u(2);
y1=u(3);y2=u(4);
th=u(5);dth=u(6);

z1=x1-epc*sin(th);
z2=x2-epc*cos(th)*dth;
dz1=z2;
```

```
z1d=t;dz1d=1;ddz1d=0;
z1e=z1-z1d;
dz1e=dz1-dz1d;

w1=y1+epc*(cos(th)-1);
w2=y2-epc*sin(th)*dth;
dw1=w2;
w1d=sin(t);dw1d=cos(t);ddw1d=-sin(t);
w1e=w1-w1d;
dw1e=dw1-dw1d;
alfa1 and beta1 must meet alfa11+beta1<=sqrt(3)/2(-alfa2-beta2-1+g)
alfa2 and beta2 must meet alfa12+beta2+1<g
v1=-alfa1*tanh(k1*z1e+l1*dz1e)-beta1*tanh(l1*dz1e)+ddz1d;
v2=-alfa2*tanh(k2*w1e+l2*dw1e)-beta2*tanh(l2*dw1e)+ddw1d;
u1b=sqrt(v1^2+(v2+g)^2);
u1=u1b+epc*dth^2;
thd=atan(-v1/(v2+g));
sys(1)=u1;
sys(2)=thd;

%%%%%%%%%%%%%%%%%    u_ctrl2
function [sys,x0,str,ts]=s_function(t,x,u,flag)
switch flag,
case 0,
     [sys,x0,str,ts]=mdlInitializeSizes;
case 3,
     sys=mdlOutputs(t,x,u);
```

```
case {1, 2, 4, 9 }
        sys = [];
otherwise
        error(['Unhandled flag = ',num2str(flag)]);
end
function [sys,x0,str,ts]=mdlInitializeSizes
sizes = simsizes;
sizes.NumDiscStates    = 0;
sizes.NumOutputs          = 1;
sizes.NumInputs           = 9;
sizes.DirFeedthrough = 1;
sizes.NumSampleTimes = 1;
sys=simsizes(sizes);
x0=[];
str=[];
ts=[0 0];
function sys=mdlOutputs(t,x,u)
x1=u(1);x2=u(2);
y1=u(3);y2=u(4);
th=u(5);dth=u(6);
thd=u(7);
dthd=u(8);
ddthd=u(9);
xite=th-thd;
dxite=dth-dthd;

c=5;k=10;
```

```
s=c*xite+dxite;

u2=-c*(dth-dthd)+ddthd-k*s;
sys(1)=u2;

%%%%%%%%%%%%Main plot
close all;
epc=0.50;
z1d=t;th=p(:,5);
x1d=z1d+epc*sin(th);

w1d=sin(t);
y1d=w1d-epc*(cos(th)-1);

figure(1);
subplot(211);
plot(t,x1d,'r',t,p(:,1),'b--','linewidth',3);
xlabel('time(s)');ylabel('x1 tracking');
legend('Ideal x1','Actual x1');
subplot(212);
plot(t,x1d-p(:,1),'k','linewidth',3);
xlabel('time(s)');ylabel('x1 tracking error');
legend('Tracking error of x1');

figure(2);
subplot(211);
plot(t,y1d,'r',t,p(:,3),'b--','linewidth',3);
```

```
xlabel('time(s)');ylabel('y1 tracking');
legend('Ideal y1','Actual y1');
subplot(212);
plot(t,y1d-p(:,3),'k','linewidth',3);
xlabel('time(s)');ylabel('y1 tracking error');
legend('Tracking error of y1');

figure(3);
subplot(211);
plot(t,thd(:,1),'r',t,p(:,5),'b--','linewidth',3);
xlabel('time(s)');ylabel('th tracking');
legend('Ideal thd','Actual th');
subplot(212);
plot(t,thd(:,1)-p(:,5),'k','linewidth',3);
xlabel('time(s)');ylabel('th tracking error');
legend('Tracking error of th');

figure(4);
subplot(211);
plot(t,dthd(:,1),'r',t,p(:,6),'b--','linewidth',3);
xlabel('time(s)');ylabel('w tracking');
legend('Ideal dthd','Actual dthd');
subplot(212);
plot(t,dthd(:,1)-p(:,6),'k','linewidth',3);
xlabel('time(s)');ylabel('w tracking error');
legend('Tracking error of dthd');
```

```
figure(5);
plot(t,ut(:,1),'k','linewidth',3);
xlabel('time(s)');ylabel('control input u1');

figure(6);
plot(t,ut(:,2),'k','linewidth',3);
xlabel('time(s)');ylabel('control input u2');

figure(7);
plot(z1d,w1d,'r','linewidth',3);
hold on;
plot(p(:,1),p(:,3),'-.k','linewidth',3);
xlabel('x');ylabel('y');
legend('ideal trajectory','practical trajectory');
```
