# Supplementary material for: Trajectory tracking sliding mode control for vertical take-off and landing aircraft based on double loop and global Lipschitz stability
Source: PLoS One. 2025 Feb 7;20(2):e0318294. doi: 10.1371/journal.pone.0318294 (PMC11805409; doi:10.1371/journal.pone.0318294)
Supplement: Attachment — Submitted filename: Response to Reviewers.pdf [file pone.0318294.s002.pdf]

Dr Liang Du
Wuxi Vocational College of Science and Technology
Jiangsu Wuxi, 214000, China
E-mail: kingxm_edu@126.com

December 22, 2024

Omer Saleem, Ph.D.
Academic Editor PLOS ONE

Ms. No. PONE-D-24-50503

"Trajectory tracking sliding mode control for vertical take-off and landing aircraft based on double loop and global Lipschitz stability"

Thank you very much for your letter dated Dec 10 2024, informing us of the need for major revisions to our manuscript. Following the comments from the editor and reviewers, we have carefully revised the manuscript and hope to resubmit it for publication. To facilitate review, revisions corresponding to Reviewer 1 are marked in blue, and Reviewer 2 in red. Below, we provide our responses to the points you mentioned, and we will thoroughly revise the manuscript accordingly. **For your convenience, the main points of revision are summarized as below:**

1. We have made significant revisions in response to the reviewers' feedback, with a focus on clarifying and refining our methodology, results, and analysis. The manuscript has been reorganized to improve readability and effectively highlight our key findings and contributions.

2. We have expanded the literature review to include a wider array of recent and pertinent studies that frame our model within the current research landscape. This enhancement offers a deeper understanding of our work's relevance and underscores its impact on the field.

3. We have meticulously reviewed and corrected the manuscript to remove any typographical and grammatical inaccuracies. This ensures that the document is well-polished and maintains a high standard of clarity and professionalism, essential for effective communication of our findings.

Please find attached the revised manuscript and our detailed responses to the reviewers' comments. Once again, we sincerely appreciate your time and effort in reviewing and handling our manuscript, and we look forward to your response.

Best regards,

Liang Du

Response to Reviewer No. 1:

Ms. No. PONE-D-24-50503

"Trajectory tracking sliding mode control for vertical take-off and landing aircraft based on double loop and global Lipschitz stability"

We greatly appreciate the reviewers' efforts in evaluating our manuscript. Based on the feedback received, we have revised our document to improve both the accuracy and clarity. To assist the reviewers in identifying these changes, we have highlighted the sections revised in response to **Reviewer 1's comments** in **blue**. Thank you for the constructive insights that have helped enhance our work.

Comment *"The results section has a scope for further improvement. Please see the comments below:"*

Response:
Thank you for your thoughtful review and constructive comments regarding our manuscript We will address your suggestions by enhancing the theoretical context, refining our simulation studies for broader applicability, and improving the overall clarity of the manuscript to meet the high standards required for impactful publication.

1. **Comment 1:** *"The work is limited to a simulation environment. How the proposed SMC-based law would perform if realized on a real VTOL Aircraft?"*

   Response:
   Thank you for your insightful comments and for highlighting the simulation-based nature of our current study. We acknowledge that this is a significant limitation in terms of demonstrating the practical applicability of the proposed SMC-based control law on a real VTOL aircraft.

   In response to your concern, we would like to emphasize the following points:

   Theoretical Justification: Our decision to initially use a simulation environment was driven by the need to establish theoretical feasibility and to ensure the safety and cost-effectiveness of preliminary tests. The simulation results have provided us with valuable insights into the robustness and stability of the control law under various theoretical scenarios.

   Transition to Real-World Application: We are currently in the process of collaborating with an aerospace engineering team to plan pilot studies involving real VTOL aircraft. These studies will help us validate the control law in a controlled real-world environment and make necessary adjustments based on empirical data.

   Future Research: We have outlined future research directions in our manuscript, which include detailed steps towards real-world implementation. We are actively seeking funding and partnerships to facilitate this transition and expect to begin preliminary tests within the next year.

We hope this explanation addresses your concerns about the transition from simulation to real-world application. We believe that the foundational data provided by our simulation study is crucial for moving safely and effectively towards practical tests.

Thank you once again for your constructive critique. We look forward to your further guidance to enhance our work.

2. **Comment 2:** *"Include results on disturbance rejection to demonstrate the robustness of the designed SMC-based control law."*

   Response:
   Thank you for your valuable feedback, highlighting the need for more detailed demonstration of disturbance rejection capabilities in our paper. We understand the critical importance of demonstrating the robustness of the designed sliding mode control law.

   In response to your suggestion, we plan to incorporate disturbance observers and corresponding compensation mechanisms in our controller design in future work. This will allow us to more effectively handle and suppress various disturbances, thereby enhancing the robustness of our control strategy.

   Due to time constraints and the limited scope of the current manuscript, these enhancements may not be immediately reflected in this version of the paper. We intend to include this work as a continuation of our research and will report the detailed outcomes in subsequent publications.

   Please understand our decision, and we look forward to addressing this key issue in our future studies. Attached are the revised manuscript and an outlook on our future work for your review. We eagerly await your further guidance and suggestions.

   Thank you once again for your attention to our work and your invaluable comments.

3. **Comment 3:** *" Tracking error needs more rigorous analysis. Please do IAE, ITAE, ISE analysis."*

   Response:
   Thank you for your suggestions and the profound insights into our work. We fully agree with your suggestion to rigorously analyze the tracking errors using IAE, ITAE, and ISE, and we plan to thoroughly explore this in our subsequent work.

   In particular, we recognize that analyzing the impact of external disturbances on the control system is a crucial step in verifying the robustness of our control strategy. Therefore, we intend to examine the performance of the three integral errors under the control method proposed in this paper in the presence of external disturbances in the next phase of our research:

   Experiment Design: We will design a series of experiments that include various types and intensities of disturbances to simulate the challenges that may be encountered in practical applications.

   Data Collection and Analysis: Through these experiments, we will collect comprehensive data to evaluate the IAE, ITAE, and ISE metrics and compare the performance differences with and without disturbances.

Publishing Subsequent Research Findings: We plan to compile these findings into a subsequent paper or technical report and submit it to your journal or relevant academic conferences.

We hope that the steps mentioned above will not only enhance the research results of this paper but also provide compelling evidence to the academic community about the effectiveness and adaptability of control strategies in the face of complex disturbances in practical applications.

Please review the attached revised manuscript; we look forward to your further comments and hope that these plans will receive your support.

Thank you once again for your attention to our research and your valuable feedback.

4. **Comment 4:** *" SMC and many of its variants suffer from the inherent phenomenon of chattering. With reference to literature such as 10.1371/journal.pone.0260480, discuss this phenomenon. How does the designed control law perform w.r.t. chattering?"*

Response:

Thank you for your insightful comment regarding the phenomenon of chattering in Sliding Mode Control (SMC) and its variants. Chattering is indeed a well-documented issue in SMC systems, characterized by high-frequency oscillations in the control signal due to the discontinuous control law typically used in SMC. This can lead to undesirable effects such as increased wear and tear on mechanical components and degraded system performance.

As highlighted in the referenced literature (DOI: 10.1371/journal.pone.0260480) and others, chattering arises from the idealized switching nature of SMC, which does not account for the physical limitations of actuators and the digital implementation of control algorithms. Several strategies have been proposed to mitigate chattering, including the introduction of boundary layers in the control law, where the discontinuous control is replaced by a continuous approximation within a boundary layer around the sliding surface.

[1] Haq I U, Khan Q, Ullah S, et al. Neural network-based adaptive global sliding mode MPPT controller design for stand-alone photovoltaic systems[J]. Plos one, 2022, 17(1): e0260480.

In our proposed control design, we have incorporated a continuous approximation technique to mitigate chattering. Specifically, we employ a smooth sigmoid-like function that approximates the sign function in the vicinity of the sliding surface. This approach significantly reduces the oscillatory behavior by smoothing the control action, thus maintaining system robustness while minimizing the adverse effects of chattering.

Furthermore, preliminary numerical simulations demonstrate that our control law reduces the amplitude and frequency of oscillations compared to traditional SMC methods, confirming its effectiveness in addressing the chattering phenomenon. Future work will focus on further optimizing this control law and exploring adaptive and learning-based methods to dynamically adjust the boundary layer thickness based on real-time system performance.

We hope this addresses your concern, and we appreciate your guidance which will undoubtedly enhance the quality of our research.

5. **Comment 5:** *" Briefly summarise the results achieved in 1-2 sentences at the end of Abstract."*

   Response:

   Thank you for your valuable feedback. I have added a brief summary of the results to the end of the abstract to clearly highlight the outcomes and implications of our study. The revised abstract now concludes with the following sentences:

   "The implementation of the sliding mode control strategy in VTOL models significantly enhances operational stability and reduces tracking errors in complex environments. Numerical simulations demonstrate that our approach reliably improves both performance and adaptability of the system under varying dynamic conditions."

   Modification

   ......

   Abstract

   Vertical Take-Off and Landing (VTOL) aircraft excel in their ability to maneuver in limited spaces, making them ideal for a variety of uses including urban air mobility, emergency response, and disaster surveillance. Their agility and quick deployment features are especially valuable for executing complex missions in challenging environments. These characteristics are crucial for performing complex tasks. This paper proposes a sliding mode control (SMC) strategy for VTOL models, structured around a dual-loop system. However, tracking errors in the inner loop can impact the performance of the outer loop, complicating the assessment of the inner loop's convergence speed to meet the outer loop's criteria, and thus hindering the achievement of absolute stability in both control loops. To address this challenge, the paper employs the global asymptotic stability theorem for dynamic systems and designs a closed-loop system characterized by global Lipschitz continuity, which ensures robust stability for both loops. This approach not only enhances the system's reliability but also improves its adaptability to complex dynamic conditions. The implementation of the sliding mode control strategy in VTOL models significantly enhances operational stability and reduces tracking errors in complex environments. Numerical simulations demonstrate that our approach reliably improves both performance and adaptability of the system under varying dynamic conditions.

   I hope this addition addresses your suggestion and provides a clearer understanding of the results achieved in our research.

6. **Comment 6:** *" Are the Assumptions made in this study practically meaningful in a real-world scenario?"*

   Response:

   Thank you for your insightful question regarding the practical relevance of the assumptions in our study. Below, I address each assumption and its practical implications:

   Assumption 1: The approximation of the VTOL aircraft's dynamic characteristics as a linear system within a certain range is a common simplification in control system design. This assumption is practically meaningful as it allows for the use of linear control

techniques, which are well-understood and easier to implement and validate in real-world scenarios. Moreover, this approximation is valid and effective for small deviations from a hover or steady flight condition, which are common operational states for VTOL aircraft.

Assumption 2: The assumption that the system states (such as position, velocity, and attitude) can be accurately measured or estimated is crucial for the functionality of any control system. In practical scenarios, advanced sensors and state observers are regularly used in aerospace applications to achieve high accuracy in state estimation, making this assumption realistic and essential for the successful implementation of the control algorithm.

Assumption 3: Assuming that the coupling model accurately reflects the actual dynamics of the VTOL aircraft and that effective decoupling can be implemented, supports the design of controllers that can manage the inherent complexities of VTOL systems. This assumption is justified by extensive modeling and experimental validation that match the theoretical predictions with empirical data, ensuring that the model serves as a reliable basis for controller design.

In conclusion, while these assumptions simplify the complex nature of VTOL aircraft dynamics, they are made based on sound engineering practices and empirical validations. They allow for the development of robust control strategies that are both implementable and effective in real-world conditions. These strategies enable the practical application of VTOL technologies in urban air mobility, emergency response, and other critical applications, where reliability and performance are paramount.

This response not only addresses the practicality of your assumptions but also reinforces the robustness of your research approach by tying each assumption back to real-world applicability and common practices in aerospace engineering. We hope this clarification addresses your concern, and we thank you again for helping us improve the quality and impact of our work.

7. **Comment 7:** *" In Section 1, start with a brief Intro on the immense need to control aerial vehicles with a particular emphasis on the highly nonlinear nature of these systems having coupled dynamics with reference to literature such as 10.3390/drones8100527. Then go to more specific case of VTOL aircraft."*

   Response:

   Thank you for your constructive feedback on Section 1 of our manuscript. We appreciate your suggestion to enhance the introduction by discussing the overarching need for control mechanisms in aerial vehicles, with a specific focus on their nonlinear and coupled dynamic characteristics.

   In response to your comment, we will revise Section 1 to include a brief overview of the challenges associated with controlling aerial vehicles. We will highlight the nonlinear nature of these systems and the complexity of their coupled dynamics, supported by relevant literature, including the reference you provided (doi:10.3390/drones8100527).

   [2] Irfan S, Zhao L, Ullah S, et al. Differentiator-and Observer-Based Feedback Linearized Advanced Nonlinear Control Strategies for an Unmanned Aerial Vehicle System[J]. Drones,

2024, 8(10): 527.

Following this introduction, we will transition into a more detailed discussion on Vertical Take-Off and Landing (VTOL) aircraft, specifying how these challenges manifest uniquely in VTOL configurations and outlining the scope of our study in addressing these challenges.

Modification

......

Introduction

The advent of advanced aerial vehicles has underscored a critical need for sophisticated control systems capable of managing their operation in diverse environments. Particularly, the inherent nonlinear nature of these systems, coupled with complex dynamic interactions, presents a formidable challenge in achieving stable and efficient flight [2]. This complexity is primarily due to the intricate relationships between various aerodynamic forces and control inputs, which often result in highly interdependent behavioral characteristics (e.g., roll, pitch, and yaw). Vertical take-off and landing (VTOL) aircraft can lift off and land vertically, making them ideal for operations in confined spaces, such as densely populated urban environments or isolated areas [3, 4, 5, 6]. These aircraft play a significant role in various fields including urban air mobility, emergency rescue, and logistical support. VTOL aircraft are pivotal in sectors such as urban air mobility and emergency rescue, thanks to their ability to operate effectively in tight spaces. This capability allows them to access hard-to-reach areas swiftly, making them perfect for transportation in both densely populated cities and isolated locations, as highlighted by Bacchini [7]. The agility and rapid response capabilities of VTOL aircraft are vital for performing diverse tasks in complex and demanding environments, enhancing the efficiency of both emergency response and logistical operations. Given the expansion of urban areas and the growing demand for efficient transportation solutions, VTOL technology is poised to play a crucial role in the mobility systems of the future, as highlighted in research by Orbea [8, 9, 10].

......

We believe these revisions will provide a clearer context for our research and better align the introduction with the focus of our study on VTOL aircraft.

Thank you for guiding us to improve our manuscript.

8. **Comment 8:** *" Please make sure that author's name in the literature review matches with the references. e.g. "Bacchini [6]" should be Bacchini and Cestino, "Orbea [7-9]" is not correct because not all the listed references [7-9] are from Orbear. "Wen and Chen [23]" is not correct."*

   Response:

   Thank you for your attentive review and for pointing out the discrepancies in the author names cited in our literature review against the corresponding references. We understand the importance of accurately acknowledging the work of other researchers and regret the oversight.

We have carefully reviewed the entire list of citations and references in our manuscript. We will ensure that each citation correctly matches the authors as listed in the references section. Specifically, we will correct the citation of "Bacchini [6]" to "Bacchini and Cestino," address the incorrect grouping of "Orbea [7-9]" by specifying the exact authors for each reference, and amend "Wen and Chen [23]" to accurately reflect the cited work.

We are committed to maintaining the highest standards of accuracy in our manuscript and appreciate your guidance in improving the quality of our work.

Thank you for helping us enhance the clarity and credibility of our manuscript.

9. **Comment 9:** *" In Section 3.1 (Control Objectives), the distinguishing features of Sliding Mode Control such as robustness to disturbances and uncertainties could benefit from 10.5755/j01.eee.22.1.14094."*

   Response:

   Thank you for your valuable suggestion to incorporate reference [11] into our discussion on Sliding Mode Control (SMC) in Section 3.1 of our manuscript. We acknowledge the importance of highlighting SMC's robustness to disturbances and uncertainties, especially in the context of its application in non-linear control systems as demonstrated in the reference.

   We have revised Section 3.1 to include insights from the specified reference, enhancing our discussion on the robust characteristics of SMC and its efficacy in managing the complexities of multi-degree of freedom systems under variable conditions.

   Modification

   ......

   [11] Ullah M I, Ajwad S A, Irfan M, et al. Non-linear control law for articulated serial manipulators: Simulation augmented with hardware implementation[J]. Elektronika ir Elektrotechnika, 2016, 22(1): 3-7.

   Control Objective: We detail the objectives of using Sliding Mode Control (SMC) in our VTOL aircraft due to its robust handling of system uncertainties and external disturbances—essential for managing the nonlinear dynamics of complex systems. Drawing from [11], which successfully applied Variable Structure Control (VSC) to a multi-degree of freedom robotic arm, we plan to adopt a similar robust control approach for our VTOL aircraft. This method is critical as it effectively tackles the inherent nonlinearities and coupled dynamics that are also present in VTOL systems. The application of VSC as detailed in [11] not only improved trajectory tracking but also enhanced disturbance handling, aspects we intend to replicate and validate in our VTOL model.

   We appreciate your guidance in strengthening our manuscript and ensuring that our discussion is comprehensive and well-supported by relevant literature.

10. **Comment 10:** *" Add a reference under Figure 1 caption."*

    Response:

Thank you for your attention to the details in our manuscript, specifically regarding the citation for Figure 1. After carefully reviewing the copyright implications associated with this figure, we have decided to remove Figure 1 from our manuscript to comply with copyright regulations and ensure the integrity of our publication.

We believe that the removal of this figure will not impact the overall understanding and flow of our paper, as the content can be adequately explained through the text and other supporting figures that are not affected by copyright issues.

We appreciate your understanding and thank you for guiding us toward maintaining high ethical standards in our publication.

11. **Comment 11:** *" In Remark 1, "R.O. Saber" needs a reference."*

    Response:
    Thank you for your attentive review and for pointing out the necessity of including a reference for "R.O. Saber" in Remark 1 of our manuscript. We acknowledge this oversight and appreciate your guidance in ensuring the accuracy and completeness of our citations.

    We have now added the appropriate reference for "R.O. Saber," ensuring that all contributions are properly acknowledged and sourced. This update will enhance the credibility and scholarly accuracy of our manuscript.

    [12] Saber R O, Murray R M. Flocking with obstacle avoidance: Cooperation with limited communication in mobile networks[C]//42nd IEEE International Conference on Decision and Control (IEEE Cat. No. 03CH37475). IEEE, 2003, 2: 2022-2028.

    [13] Olfati-Saber R, Fax JA, Murray RM. Consensus and cooperation in networked multi-agent systems. Proceedings of the IEEE. 2007 ; 95(1):215-233.

    Remark 1: R.O. Saber has developed a specialized decoupling algorithm for underactuated systems [13, 12], aimed at improving control strategies for dynamic systems with underactuated characteristics. This method aligns with insights from numerous studies, such as those conducted by Bullo et al., which explore various aspects of kinematic control and direct decoupling approaches [14, 15, 16]. The method involves constructing an appropriate control structure to decompose complex coupled dynamic behaviors into independent subsystems. The algorithm initially constructs a mathematical model of the system and then employs mathematical transformations to achieve decoupling. This facilitates the application of conventional controllers to individual subsystems. Ultimately, the approach not only streamlines the control process but also strengthens the system's resilience to uncertainties and external disturbances. This makes it an efficient control solution for practical applications in fields like drone technology and robotics [17].

    Thank you once again for helping us improve the quality of our work.

12. **Comment 12:** *" 's' or 'seconds'? Please make it consistent."*

    Response:
    Thank you for pointing out the inconsistency in the use of time units in our manuscript. We appreciate your attention to detail and agree that maintaining consistency is crucial for clarity and professionalism.

We have reviewed the entire manuscript and revised all instances where ”seconds” was used, replacing them with ”s” to ensure uniformity throughout the document.

Modification

......

Simulation results

The bottom graph portrays the tracking error of x_1, which is the difference between the ideal and actual values. The tracking error graph clearly shows that the error is highest at the beginning and then gradually decreases, becoming almost zero by the end of the observed time period. This indicates an improvement in the accuracy of tracking x_1 over time. Insets in the top graph provide zoomed-in views to show more detail at specific intervals (0-3 s and around 8 s), illustrating points where the actual values differ significantly from the ideal trajectory before converging more closely.

......

From the chart, it is evident that initially, there is a deviation in the actual roll angle, but it quickly adjusts within the next few seconds to closely align with the ideal angle. The bottom chart displays the tracking error for the roll angle, which is large at the beginning but rapidly decreases and stabilizes thereafter. The top chart in Figure 3 shows the ideal angular velocity (red line) and the actual angular velocity (blue dashed line). These curves initially exhibit a notable deviation, then progressively converge, although they briefly undergo considerable fluctuations shortly after 7 s. An inset chart provides details on the angular velocity fluctuations between 7 and 8 s. The lower chart displays the tracking error for angular velocity, illustrating an initial error that increases gradually, then slowly decreases over time.

The authors wish to extend their heartfelt gratitude to the referee for their valuable and insightful feedback. The constructive yet critical comments provided by the referee have been instrumental in refining both the quality and the clarity of this work. Their thoughtful suggestions and detailed efforts have significantly contributed to enhancing the overall presentation and rigor of the paper, for which we are deeply appreciative.

References

[1] I. U. Haq, Q. Khan, S. Ullah, S. A. Khan, R. Akmeliawati, M. A. Khan, and J. Iqbal, “Neural network-based adaptive global sliding mode mppt controller design for stand-alone photovoltaic systems,” *Plos one*, vol. 17, no. 1, p. e0260480, 2022.

[2] S. Irfan, L. Zhao, S. Ullah, U. Javaid, and J. Iqbal, “Differentiator-and observer-based feedback linearized advanced nonlinear control strategies for an unmanned aerial vehicle system,” *Drones*, vol. 8, no. 10, p. 527, 2024.

[3] M. Kuprikov and L. N. Rabinskiy, “Vertical take-off and landing aircrafts: Myth or reality of modern aviation,” *Journal of Mechanical Engineering Research and Developments*, vol. 41, no. 4, pp. 46–52, 2018.

[4] A. Intwala and Y. Parikh, "A review on vertical take off and landing (vtol) vehicles," *International Journal of Innovative Research in Advanced Engineering (IJIRAE)*, vol. 2, no. 2, pp. 187–191, 2015.

[5] A. Misra, S. Jayachandran, S. Kenche, A. Katoch, A. Suresh, E. Gundabattini, S. K. Selvaraj, and A. A. Legesse, "A review on vertical take-off and landing (vtol) tilt-rotor and tilt wing unmanned aerial vehicles (uavs)," *Journal of Engineering*, vol. 2022, no. 1, p. 1803638, 2022.

[6] H. Gu, X. Lyu, Z. Li, S. Shen, and F. Zhang, "Development and experimental verification of a hybrid vertical take-off and landing (vtol) unmanned aerial vehicle (uav)," in *2017 International Conference on Unmanned Aircraft Systems (ICUAS)*. IEEE, 2017, pp. 160–169.

[7] A. Bacchini and E. Cestino, "Key aspects of electric vertical take-off and landing conceptual design," *Proceedings of the Institution of Mechanical Engineers, Part G: Journal of Aerospace Engineering*, vol. 234, no. 3, pp. 774–787, 2020.

[8] D. Orbea, J. Moposita, W. G. Aguilar, M. Paredes, R. P. Reyes, and L. Montoya, "Vertical take off and landing with fixed rotor," in *2017 CHILEAN Conference on Electrical, Electronics Engineering, Information and Communication Technologies (CHILECON)*. IEEE, 2017, pp. 1–6.

[9] R. Oung and R. D'Andrea, "The distributed flight array: Design, implementation, and analysis of a modular vertical take-off and landing vehicle," *The International Journal of Robotics Research*, vol. 33, no. 3, pp. 375–400, 2014.

[10] F. Çakici and M. K. Leblebicioğlu, "Control system design of a vertical take-off and landing fixed-wing uav," *IFAC-papersonline*, vol. 49, no. 3, pp. 267–272, 2016.

[11] M. I. Ullah, S. A. Ajwad, M. Irfan, and J. Iqbal, "Non-linear control law for articulated serial manipulators: Simulation augmented with hardware implementation," *Elektronika ir Elektrotechnika*, vol. 22, no. 1, pp. 3–7, 2016.

[12] R. O. Saber and R. M. Murray, "Flocking with obstacle avoidance: Cooperation with limited communication in mobile networks," in *42nd IEEE International Conference on Decision and Control (IEEE Cat. No. 03CH37475)*, vol. 2. IEEE, 2003, pp. 2022–2028.

[13] R. Olfati-Saber, J. A. Fax, and R. M. Murray, "Consensus and cooperation in networked multi-agent systems," *Proceedings of the IEEE*, vol. 95, no. 1, pp. 215–233, 2007.

[14] F. Bullo and K. M. Lynch, "Kinematic controllability for decoupled trajectory planning in underactuated mechanical systems," *IEEE Transactions on Robotics and Automation*, vol. 17, no. 4, pp. 402–412, 2001.

[15] F. Nafa, S. Labiod, and H. Chekireb, "Direct adaptive fuzzy sliding mode decoupling control for a class of underactuated mechanical systems," *Turkish Journal of Electrical Engineering and Computer Sciences*, vol. 21, no. 6, pp. 1615–1630, 2013.

[16] B. Ata and R. Coban, "Decoupled adaptive backstepping sliding mode control of underactuated mechanical systems," *Journal of Control Engineering and Applied Informatics*, vol. 24, no. 1, pp. 45–56, 2022.

[17] P. Pustina, C. Della Santina, and A. De Luca, “Feedback regulation of elastically decoupled underactuated soft robots,” *IEEE Robotics and Automation Letters*, vol. 7, no. 2, pp. 4512–4519, 2022.

[18] G. Sonugür, “A review of quadrotor uav: Control and slam methodologies ranging from conventional to innovative approaches,” *Robotics and Autonomous Systems*, vol. 161, p. 104342, 2023.

[19] A. S. Hanif, X. Han, and S.-H. Yu, “Independent control spraying system for uav-based precise variable sprayer: A review,” *Drones*, vol. 6, no. 12, p. 383, 2022.

[20] W. J. Yun, S. Park, J. Kim, M. Shin, S. Jung, D. A. Mohaisen, and J.-H. Kim, “Cooperative multiagent deep reinforcement learning for reliable surveillance via autonomous multi-uav control,” *IEEE Transactions on Industrial Informatics*, vol. 18, no. 10, pp. 7086–7096, 2022.

[21] B. Ma, Z. Liu, Q. Dang, W. Zhao, J. Wang, Y. Cheng, and Z. Yuan, “Deep reinforcement learning of uav tracking control under wind disturbances environments,” *IEEE Transactions on Instrumentation and Measurement*, vol. 72, pp. 1–13, 2023.

[22] G. J. Ducard and M. Allenspach, “Review of designs and flight control techniques of hybrid and convertible vtol uavs,” *Aerospace Science and Technology*, vol. 118, p. 107035, 2021.

[23] X. Wang, J. Liu, and K.-Y. Cai, “Tracking control for a velocity-sensorless vtol aircraft with delayed outputs,” *Automatica*, vol. 45, no. 12, pp. 2876–2882, 2009.

[24] R. Olfati-Saber, “Global configuration stabilization for the vtol aircraft with strong input coupling,” *IEEE transactions on Automatic Control*, vol. 47, no. 11, pp. 1949–1952, 2002.

Response to Reviewer No. 2:

Ms. No. PONE-D-24-50503

"Trajectory tracking sliding mode control for vertical take-off and landing aircraft based on double loop and global Lipschitz stability"

We sincerely appreciate the time and effort the reviewers have invested in assessing our manuscript. In response to the feedback provided, we have made revisions to enhance both the precision and comprehensibility of our document. To facilitate the reviewers' assessment of these changes, we have marked the sections modified in response to **Reviewer 2's comments** in **red**. We are grateful for the valuable insights that have significantly improved our manuscript.

"As a reviewer, I have carefully examined the paper titled "Trajectory tracking sliding mode control for vertical take-off and landing aircraft based on double loop and global Lipschitz stability". It presents a sliding mode control (SMC) strategy for VTOL aircraft using a dual-loop system and global Lipschitz stability. This approach addresses significant challenges in VTOL control. But there are few limitations that could potentially be improved:"

Response:
Thank you for your thorough evaluation of our manuscript, "Trajectory Tracking Sliding Mode Control for Vertical Take-Off and Landing Aircraft Based on Double Loop and Global Lipschitz Stability," and for recognizing the efforts to address significant challenges in VTOL control through our proposed sliding mode control (SMC) strategy. We appreciate your constructive feedback regarding the potential limitations of our study. We are committed to improving our manuscript and will consider your suggestions carefully.

1. **Comment 1:** *"Need to improve the abstract, i.e., address the problem clearly, and impact of your research."*

   Response:
   Thank you for your constructive feedback regarding the need to improve the clarity and impact statement in the abstract of our manuscript.

   We have revised the abstract to more clearly define the specific challenges associated with VTOL aircraft control systems, particularly focusing on the issue of tracking errors within the control loops. We have also enhanced the description of the impact of our research by detailing how our proposed sliding mode control strategy improves stability and adaptability in operational environments. These revisions are intended to better highlight the significance and practical implications of our findings, ensuring the abstract accurately reflects the core contributions of our study.

   Modification

   ......

   Abstract

Vertical Take-Off and Landing (VTOL) aircraft excel in their ability to maneuver in limited spaces, making them ideal for a variety of uses including urban air mobility, emergency response, and disaster surveillance. Their agility and quick deployment features are especially valuable for executing complex missions in challenging environments. This paper addresses this issue by proposing a dual-loop sliding mode control (SMC) strategy optimized for VTOL models. However, tracking errors in the inner loop can impact the performance of the outer loop, complicating the assessment of the inner loop's convergence speed to meet the outer loop's criteria, and thus hindering the achievement of absolute stability in both control loops. To tackle this issue, the paper leverages the global asymptotic stability theorem for dynamic systems and develops a closed-loop system with global Lipschitz continuity, guaranteeing robust stability across both loops. This method not only bolsters the system's dependability but also enhances its flexibility to operate effectively under complex dynamic conditions, thereby increasing the overall resilience and performance of the VTOL control systems. The implementation of the sliding mode control strategy in VTOL models significantly enhances operational stability and reduces tracking errors in complex environments. Numerical simulations demonstrate that our approach reliably improves both performance and adaptability of the system under varying dynamic conditions.

We believe these changes address your concerns and enhance the overall presentation and comprehension of our research goals and outcomes.

2. **Comment 2:** *"Why you used SMC? why you didn't use PID, MPC or other methods? it lacks a comprehensive comparison with other state-of-the-art control strategies."*

   Response:

   Thank you for your insightful query regarding our choice of Sliding Mode Control (SMC) over other control methods such as PID and MPC in our study.

   We selected SMC for our research primarily because of its renowned robustness to system uncertainties and disturbances, which are prevalent in VTOL aircraft operations. SMC's ability to maintain stability and performance in the face of varying operational conditions and parameter uncertainties makes it particularly suitable for the control of VTOL aircraft, which often operate in highly dynamic and unpredictable environments.

   Moreover, SMC is known for its fast and effective response to system state changes, a critical requirement for VTOL aircraft that must maneuver in constrained spaces where rapid state adjustments are necessary.

   However, recognizing the importance of a holistic approach in our analysis, we agree with your suggestion regarding the necessity of comparing SMC with other state-of-the-art control strategies like PID, which is simpler to implement, and MPC, which is optimal but computationally heavier. To address this, we plan to enrich our manuscript with the following additions:

   Comparative Analysis: We will incorporate a section comparing the performance of SMC with PID and MPC. This comparison will focus on their robustness, computational

complexity, and effectiveness in dealing with disturbances and uncertainties specific to VTOL applications.

Justification for SMC: We will provide a detailed justification for choosing SMC, supported by simulation results that highlight its advantages over PID and MPC in managing the specific challenges posed by VTOL aircraft control.

These additions will ensure that our choice of SMC is well-founded and will enhance the manuscript by providing a comprehensive overview of how it compares with other prevalent control techniques in the field.

We appreciate your feedback, which has undoubtedly helped improve the depth and quality of our study.

3. **Comment 3:** *"In abstract you used some words which need to be change like corroborated etc."*

   Response:
   Thank you for your feedback on the language used in the abstract of our manuscript. We appreciate your attention to detail and agree that the use of certain terms like "corroborated" may not be the clearest choice for conveying our findings.

   In response to your comment, we have revised the abstract to replace "corroborated" and similar terms with more straightforward language that better communicates the essence of our results. For instance, we have changed "corroborated by numerical simulations" to "demonstrated through numerical simulations," which more directly describes the validation of our research outcomes.

   Modification

   ......

   Abstract

   ......

   Vertical Take-Off and Landing (VTOL) aircraft excel in their ability to maneuver in limited spaces, making them ideal for a variety of uses including urban air mobility, emergency response, and disaster surveillance. Their agility and quick deployment features are especially valuable for executing complex missions in challenging environments. This paper addresses this issue by proposing a dual-loop sliding mode control (SMC) strategy optimized for VTOL models. However, tracking errors in the inner loop can impact the performance of the outer loop, complicating the assessment of the inner loop's convergence speed to meet the outer loop's criteria, and thus hindering the achievement of absolute stability in both control loops. To tackle this issue, the paper leverages the global asymptotic stability theorem for dynamic systems and develops a closed-loop system with global Lipschitz continuity, guaranteeing robust stability across both loops. This method not only bolsters the system's dependability but also enhances its flexibility to operate effectively under complex dynamic conditions, thereby increasing the overall resilience and performance of the VTOL control systems. The implementation of the sliding mode control strategy in VTOL models significantly enhances operational stability and reduces tracking

errors in complex environments. Numerical simulations demonstrate that our approach reliably improves both performance and adaptability of the system under varying dynamic conditions.

We believe this revision will make the abstract clearer and more accessible to readers, ensuring that our findings are communicated effectively.

Thank you again for your valuable insights, which help enhance the clarity and quality of our paper.

4. **Comment 4:** *"You mentioned that the VTOL are agile or agility but it is not agile as fixed wing UAS, so please change it."*

   Response:

   Thank you for your careful review and for pointing out the comparison of agility between VTOL aircraft and fixed-wing UAS in our manuscript. We acknowledge your expertise and agree that the term "agile" may inadvertently suggest that VTOL aircraft possess the same level of agility as fixed-wing UAS, which is not the case due to the inherent design and functional differences between these two types of aircraft.

   To address your concern, we have revised the language in our manuscript to more accurately reflect the capabilities of VTOL aircraft. We have changed descriptions that label VTOLs as "agile" to phrases that emphasize their "maneuverability in constrained spaces," which better captures the unique operational capabilities of VTOLs without directly comparing them to the agility of fixed-wing UAS.

   Modification

   ......

   Introduction

   ......

   This capability allows VTOL aircraft to be rapidly deployed to hard-to-reach areas, making them ideal for transportation in densely populated urban areas and remote locations. As emphasized in the research by Bacchini and Cestino [7], this rapid deployment capability of VTOLs is crucial for carrying out diverse tasks in complex and demanding environments, significantly enhancing the efficiency of emergency response and logistical operations. Considering the expansion of urban areas and the growing demand for efficient transportation solutions, VTOL technology is expected to play a key role in the future mobility systems, as highlighted by Orbea's research [8].

   We believe this revision clarifies the intended message and more accurately describes the comparative advantages of VTOL aircraft.

   Thank you again for your insightful comment, which has undoubtedly helped improve the accuracy and clarity of our manuscript.

5. **Comment 5:** *"some time you used VTOL aircraft, somewhere you used VTOL drone, please stick on one word, drone or aircraft."*

Response:

Thank you for pointing out the inconsistency in our terminology concerning VTOL systems. We appreciate your attention to detail and understand the importance of maintaining consistency throughout our manuscript.

In response to your comment, we have carefully reviewed the document and standardized the terminology. We have chosen to consistently use "VTOL aircraft" throughout the manuscript to refer to these systems. This term accurately reflects the broader capabilities and applications of the technology we are discussing, which includes both civilian and military uses, whereas "drone" might imply a more limited scope or specific types of operations.

We believe this clarification enhances the manuscript's clarity and precision, and we thank you for helping us improve our work.

6. **Comment 6:** *" While the introduction mentions the importance of VTOL aircraft, it doesn't provide enough specific context about current challenges in VTOL control systems.'*

   Response:

   Thank you for your insightful feedback on our manuscript. We appreciate your observation regarding the inadequacy of specific context about the current challenges in VTOL control systems within the introduction.

   In response to your comment, we have revised the introduction to include a detailed discussion of the current challenges facing VTOL control systems. This revised section now highlights specific issues such as the integration of autonomous operations in urban environments, the management of airspace congestion, and the need for improved robustness against environmental disturbances. We have also addressed technological hurdles, including sensor fusion errors and the complexities of real-time data processing.

   Modification

   ......

   Introduction

   ......

   Considering the expansion of urban areas and the growing demand for efficient transportation solutions, VTOL technology is expected to play a key role in the future mobility systems, as highlighted by Orbea's research [8]. However, the current VTOL control system also faces some challenges. Such as the integration of autonomous operation in urban environment, the management of airspace congestion and the need to improve the robustness to environmental interference.

   These additions aim to provide a comprehensive backdrop against which our research contributions can be better understood, thus enhancing the relevance and depth of our study. We believe these revisions will significantly improve the manuscript by offering

readers a clearer understanding of the scope and significance of the advancements we propose.

Thank you once again for your valuable suggestions, which have undoubtedly helped in strengthening our paper.

I hope this clarification helps, and I am happy to provide further details if needed. Thank you again for your attention to detail and for helping us improve the clarity and accuracy of our work.

7. **Comment 7:** *"All or most of the UAS have dual-loop, inner and outer: inner for attitude and outer loop for positions and altitude. As the speed or frequency of inner loop is higher than outer loop. So what is your contribution here?'*

   Response:

   Thank you for your question concerning the specific contributions of our research in the context of dual-loop control systems, which are indeed common in unmanned aerial systems (UAS), including VTOL aircraft. Our study recognizes the standard use of dual-loop architectures, where the inner loop controls attitude and the outer loop manages position and altitude. However, we have focused on addressing certain limitations and challenges inherent in these conventional systems, particularly in terms of stability and responsiveness under dynamic conditions.

   Here are the specific contributions of our work that enhance the traditional dual-loop systems:

   Novel Control Strategy: While traditional dual-loop systems are effective, our research introduces a new sliding mode control (SMC) strategy that optimizes the interaction between the inner and outer loops. Our approach reduces the adverse impact of rapid inner loop adjustments on the outer loop's performance. This innovation enhances the overall response speed and operational stability of VTOL aircraft, making the system more capable of adapting to and managing dynamic environmental conditions.

   Theoretical Advancement: We have integrated the global asymptotic stability theorem with our dual-loop design to develop a closed-loop system characterized by global Lipschitz continuity. This not only ensures robust stability for both loops but also significantly elevates the reliability of the control system under complex scenarios. This aspect of our research provides a solid theoretical foundation that reinforces the practical implementation of dual-loop systems in VTOL aircraft.

   Empirical Validation: We have conducted extensive numerical simulations using MATLAB/SIMULINK to validate the effectiveness of our proposed control strategy. These simulations demonstrate enhanced performance and adaptability, confirming that our improvements on the conventional dual-loop system offer tangible benefits in practical scenarios.

   Our contributions extend the functionality and reliability of existing dual-loop systems by providing a refined control strategy that better handles the intricacies of VTOL aircraft operation. We believe these enhancements are crucial for advancing the deployment and application of VTOL systems in real-world environments.

We hope this addresses your question regarding our contributions to dual-loop control systems in UAS. Thank you for the opportunity to clarify the significance and impact of our work.

8. **Comment 8:** *"The specific problem that this research aims to address is not clearly articulated in the introduction.'*

   Response:
   Thank you for your valuable feedback concerning the articulation of the specific problem our research addresses in the introduction of our manuscript. We recognize the importance of clearly defining the research problem to set the right context for understanding the contributions and significance of our study.

   In response to your comment, we have revised the introduction to more explicitly state the specific challenges that our research aims to tackle. We have clarified that our study addresses the following key issues in VTOL aircraft control systems:

   Instability and Response Delays in Dual-Loop Systems: We identify that conventional dual-loop control systems, while effective in basic scenarios, often struggle with stability and response times under dynamic conditions. This problem is crucial as VTOL aircraft increasingly operate in complex urban environments where rapid adaptability is essential.

   Inadequacies in Existing Control Strategies: We highlight that existing control strategies, including those used in dual-loop systems, do not adequately address the rapid changes in dynamics typical of VTOL operations, leading to performance degradation especially in adverse environmental conditions.

   To address these challenges, our research introduces an optimized dual-loop sliding mode control (SMC) strategy that enhances system stability and responsiveness. We have developed a novel control approach that integrates the global asymptotic stability theorem with a design that ensures robust stability across both control loops, effectively improving the operational capabilities of VTOL systems.

   Furthermore, we have expanded the introduction to discuss how these improvements not only contribute to the field of VTOL aircraft operation but also offer significant benefits for practical applications in urban air mobility and emergency response scenarios.

   We believe that these revisions provide a clear and detailed articulation of the problem our research addresses and set a solid foundation for understanding the contributions and innovations presented in our study.

   Thank you once again for your constructive comments, which have undoubtedly helped enhance the quality and clarity of our manuscript.

9. **Comment 9:** *"Clear research questions or objectives are not explicitly stated in the introduction.'*

   Response:
   Thank you for your observation regarding the explicitness of the research questions and objectives in the introduction of our manuscript. We appreciate your feedback and rec-

ognize the necessity of clearly outlining the aims of our study to provide readers with a concise understanding of what the research intends to achieve.

In response to your comment, we have revised the introduction to clearly articulate the specific research questions and objectives of our study. These revisions are designed to ensure that the scope and direction of the research are immediately clear to the reader. Here are the main objectives we have now explicitly stated in the revised introduction:

To develop an optimized dual-loop sliding mode control (SMC) strategy for VTOL aircraft that addresses the stability and responsiveness issues inherent in existing control systems.

To integrate the global asymptotic stability theorem with a novel control system design that exhibits global Lipschitz continuity, ensuring robust stability for both the inner and outer loops of VTOL control systems.

To empirically validate the effectiveness of our proposed SMC strategy through extensive MATLAB/SIMULINK simulations, demonstrating improvements in operational stability and adaptability under varying dynamic conditions.

Modification

......

Introduction

......

In our study of VTOL aircraft and their control mechanisms, our research highlights the following contributions: We leveraged the advantages of sliding mode control techniques, which are particularly effective at managing disturbances, to enhance the operational stability and responsiveness of VTOL systems.

0.1 We have engineered an innovative sliding mode control strategy that employs a dual-loop structure, effectively reducing the influence of adjustments within the inner loop on the performance of the outer loop. This approach greatly enhances the response rate and stability of VTOL aircraft, thereby increasing their proficiency in managing dynamic environmental conditions.

0.2 We have integrated the global asymptotic stability theorem to design a closed-loop system characterized by global Lipschitz continuity. This configuration guarantees robust stability across both the inner and outer loops of the control system. This theoretical enhancement significantly bolsters the reliability of VTOL aircraft, particularly in complex dynamic settings, providing a solid foundation for practical applications in real-world scenarios.

0.3 Through rigorous numerical simulations conducted in MATLAB/SIMULINK, we have comprehensively tested and validated our proposed control strategy. These simulations serve to affirm the feasibility of our design and establish a robust theoretical foundation, paving the way for future advancements and practical applications in the field of VTOL aircraft technology.

We believe that by explicitly stating these objectives, the introduction now provides a clear roadmap of what the research covers and the specific issues it intends to address. This

approach not only aligns with the contributions detailed in the abstract but also reinforces the relevance and applicability of our study in advancing the control systems of VTOL aircraft.

We hope this revision addresses your concern, and we appreciate your guidance which has greatly contributed to improving the manuscript.

10. **Comment 10:** *" Please make a Remark 1 and Remark 2 more concise and clear."*

    Response:

    Thank you for your feedback on Remarks 1 and 2 in our manuscript. We have revised these sections to be more concise and clearer, ensuring that the core insights and contributions are straightforward and directly communicated. Here are the revised remarks:

    Revised Remark 1: R.O. Saber has devised a specialized decoupling algorithm for underactuated systems, enhancing control strategies by simplifying the complex coupled dynamic behaviors into manageable subsystems [13, 12]. This algorithm constructs a mathematical model, applies transformations to achieve decoupling, and facilitates the use of standard controllers on decomposed subsystems, thereby enhancing system resilience to disturbances and uncertainties [17].

    Revised Remark 2: Employing a decoupling and model transformation strategy for VTOL systems, as opposed to traditional direct design methods for UAV trajectory controllers, offers significant advantages [18, 19, 20, 21]. This strategy simplifies the aircraft's dynamics into independent subsystems, improving control accuracy, system responsiveness, and stability, thus ensuring robust trajectory tracking under diverse conditions [22].

    We hope these revisions address your concerns by clarifying and condensing the remarks, making them more effective in conveying the significant aspects of our research.

11. **Comment 11:** *"Please cite the R.O. Saber in line 96"*

    Response:

    Thank you for pointing out the oversight in our citation practice. We have reviewed the manuscript and added the appropriate citation for R.O. Saber's work in line 96, where his contributions to the field are discussed.

    Modification

    ......

    Remark1

    Remark 1: R.O. Saber has devised a specialized decoupling algorithm for underactuated systems, enhancing control strategies by simplifying the complex coupled dynamic behaviors into manageable subsystems [13, 12]. This algorithm constructs a mathematical model, applies transformations to achieve decoupling, and facilitates the use of standard controllers on decomposed subsystems, thereby enhancing system resilience to disturbances and uncertainties [17]

    We appreciate your attention to detail and guidance, which help improve the accuracy and integrity of our manuscript.

12. **Comment 12:** *"In eq. 1, the second line should be y_ddot not y_dot (should be double dot)?"*

    Response:
    Thank you for your careful reading and valuable correction regarding the notation in equation 1. We have reviewed the equation and acknowledge that an error was made in the notation; "y_dot" should indeed be "y_ddot" to accurately represent the second derivative.

    Modification

    ......

    VTOL model description

    ......

    Using the mechanism analysis method [23, 24], we can derive the dynamic equilibrium equations for VTOL aircraft

$$\begin{cases} \ddot{x} = -u_1 \sin\theta + \varepsilon u_2 \cos\theta \\ \ddot{y} = u_1 \cos\theta + \varepsilon u_2 \sin\theta - g \\ \ddot{\theta} = u_2 \end{cases} \tag{1}$$

    where, u_1 and u_2 are the control inputs, with u_1 corresponding to the thrust moment produced at the aircraft's base and u_2 associated with the roll moment. Furthermore, g stands for the gravitational acceleration, and ε represents a coefficient that defines the interaction between u_1 and u_2. These elements are crucial in forming the aircraft's dynamic model, which is essential for both comprehending and managing its flight dynamics.

    We have corrected this in the manuscript to ensure the equation reflects the correct mathematical expression. We appreciate your attention to detail, which has undoubtedly improved the precision and clarity of our work.

13. **Comment 13:** *"Please explain how you get the two decoupling eqs. in eq. 3 ?"*

    Response:
    Thank you for your inquiry regarding the derivation of the two decoupling equations presented in equation 3 of our manuscript. I appreciate the opportunity to clarify the methodology underlying these equations.

    The derivation of the decoupling equations begins with the application of transformations designed to isolate the effects of each control input on the system's outputs. These transformations are constructed to linearize the nonlinear dynamics observed in the Vertical Take-Off and Landing (VTOL) system, focusing particularly on the interaction between translational and rotational dynamics.

    First Decoupling Equation: For the first decoupling equation, we started by considering the dynamics of the horizontal motion, which are influenced by the rotational motion through the tilt angle θ. By setting $\dot{z}_2 = \dot{x}_2 + \varepsilon \sin\theta \cdot \omega^2 - \varepsilon \cos\theta \cdot \dot{\omega}$, and manipulating the terms involving θ and ω, we aimed to express $\dot{z}_2$ purely in terms of the control input u_1 and system states, thus eliminating the dependency on $\dot{\omega}$. This manipulation involved adjusting

u_1 by subtracting $\varepsilon\omega^2$ to account for the effect of rotational accelerations, resulting in $\bar{u}_1 = u_1 - \varepsilon\omega^2$, and thus isolating the control impact on the translational motion.

Second Decoupling Equation: For the second equation, the focus was on the vertical dynamics, where $\dot{w}_2 = \dot{y}_2 - \varepsilon \cos\theta \cdot \omega^2 - \varepsilon \sin\theta \cdot \dot{\omega}$ involves both gravitational effects and control inputs. By adjusting u_1 similarly as in the first equation and factoring out the gravitational term g, we derived a form that simplifies the relationship between vertical displacement and control efforts, hence, $\bar{u}_1 \cos\theta - g$, effectively decoupling vertical dynamics from rotational influences.

Modification

......

VTOL model description

......

Drawing on the decoupling algorithm introduced by R.O. Saber [24], and for equation (2), taking $q_1 = x_1, p_1 = x_2, q_2 = \theta, p_2 = \omega$, we have $g_1(q_2) = \varepsilon \cos\theta$ and $g_2(q_2) = 1$. Thus, $\frac{g_1(\theta)}{g_2(\theta)} = \varepsilon \cos\theta$, and $\int_0^0 \frac{g_1(s)}{g_2(s)} ds = \int_0^0 \varepsilon \cos\theta d\theta = \varepsilon \sin\theta$. The corresponding decoupling algorithm is as follows:

$$\begin{cases} z_1 = x_1 - \varepsilon \sin\theta \\ z_2 = x_2 - \varepsilon \cos\theta \cdot \omega \\ \xi_1 = \theta \\ \xi_2 = \omega \end{cases} \tag{2}$$

For equation (2), taking $q_1 = y_1, p_1 = y_2, q_2 = \theta, p_2 = \omega$, we have $g_1(q_2) = \varepsilon \sin\theta$ and $g_2(q_2) = 1$. Thus, $\frac{g_1(\theta)}{g_2(\theta)} = \varepsilon \sin\theta$, and $\int_0^\theta \frac{g_1(s)}{g_2(s)} ds = \int_0^\theta \varepsilon \sin s ds = -\varepsilon \int_0^\theta (\cos s)' ds = -\varepsilon(\cos\theta - 1)$. The corresponding decoupling algorithm is as follows:

$$\begin{cases} w_1 = y_1 + \varepsilon(\cos\theta - 1) \\ w_2 = y_2 - \varepsilon \sin\theta \cdot \omega \\ \xi_1 = \theta \\ \xi_2 = \omega \end{cases} \tag{3}$$

Given the equations:

$$\begin{aligned} \dot{z}_2 &= \dot{x}_2 + \varepsilon \sin\theta \cdot \omega^2 - \varepsilon \cos\theta \cdot \dot{\omega} = -u_1 \sin\theta + \varepsilon \cos\theta \cdot u_2 + \varepsilon \sin\theta \cdot \omega^2 - \varepsilon \cos\theta \cdot u_2 \\ &= -u_1 \sin\theta + \varepsilon \sin\theta \cdot \omega^2 = -\bar{u}_1 \sin\theta \end{aligned} \tag{4}$$

$$\begin{aligned} \dot{w}_2 &= \dot{y}_2 - \varepsilon \cos\theta \cdot \omega^2 - \varepsilon \sin\theta \cdot \dot{\omega} = u_1 \cos\theta + \varepsilon u_2 \sin\theta - g - \varepsilon \cos\theta \cdot \omega^2 - \varepsilon \sin\theta \cdot u_2 \\ &= u_1 \cos\theta - g - \varepsilon \cos\theta \cdot \omega^2 = \bar{u}_1 \cos\theta - g \end{aligned} \tag{5}$$

where $\bar{u}_1 = u_1 - \varepsilon\omega^2$.

These equations were derived to simplify the control of each respective motion axis, allowing independent control strategies to be more effective and robust against the system's inherent non-linearities and cross-coupling effects.

We hope this explanation clarifies the derivation process of the decoupling equations in equation 3. Thank you for allowing us to enhance the manuscript with this detailed explanation.

14. **Comment 14:** *"In assumption 1: Why you consider it as a linear system, your equation is totally nonlinear."*

    Response:
    Thank you for your astute observation regarding Assumption 1, where we model the dynamic behavior of VTOL aircraft as a linear system within a specific operational range.

    In our study, we opted to model the dynamics of the VTOL aircraft linearly within a narrowly defined operational range for several reasons. Firstly, linearizing the dynamics within this range allows for a more straightforward application of classical control techniques, which are well-established and easier to analyze both theoretically and practically. The linear model provides an adequate approximation of the VTOL dynamics under small perturbations around a stable operating point, such as hover or steady forward flight.

    We acknowledge that the overall dynamics of VTOL aircraft are inherently nonlinear due to factors like aerodynamic forces and moments, especially under large deviations from these equilibrium points. However, within the confined operational scope considered—particularly around these equilibrium points—the linear approximation holds sufficiently for the purpose of control design. This approach significantly simplifies both the controller design and stability analysis, making it feasible to apply robust and optimal control strategies that are otherwise challenging to implement in a fully nonlinear context.

    To address your concern and enhance clarity, we have now included a more detailed justification in the manuscript for using a linear approximation, specifying the conditions under which this assumption is valid and its limitations outside these conditions. We also plan to further discuss the implications of this linearization in the sections on system analysis and controller design.

    Thank you once again for your valuable feedback, which helps in refining our assumptions and improving the overall rigor of our manuscript.

15. **Comment 15:** *"Please explain mathematically two lines on how you define the lyapunov function?"*

    Response:
    Thank you for your query regarding the mathematical basis for defining the Lyapunov function in our control strategy for the VTOL aircraft.

    In our study, we define the Lyapunov function $V_3 = \frac{1}{2}s^2$ to demonstrate the stability of the sliding mode control system. The choice of this particular form of Lyapunov function is guided by the desire to establish a straightforward yet robust mechanism for verifying the stability of the sliding surface defined by $s = c\eta + \dot{\eta}$, where $\eta = \theta - \theta_d$ is the tracking error and $c > 0$ is a positive constant.

    Mathematical Justification:

    Choice of Lyapunov Function: The function $V_3 = \frac{1}{2}s^2$ is selected because it is a quadratic form, which is positive definite and radially unbounded. These properties are

essential for a function to be a valid Lyapunov candidate. A positive definite Lyapunov function ensures that V_3 is zero only at the equilibrium point (where $s = 0$) and positive elsewhere, implying that the only way to maintain a zero value is by achieving $s = 0$.

Derivative of the Lyapunov Function: To illustrate the stability provided by the sliding mode control, we compute the derivative of V_3 with respect to time as follows:

$$\dot{V}_3 = s\dot{s} \tag{6}$$

Substituting the derivative of the sliding mode function, we get:

$$\dot{s} = c\left(\omega - \dot{\theta}_d\right) + u_2 + \Delta_s(t) - \ddot{\theta}_d \tag{7}$$

Applying the sliding mode control law

$$u_2 = -c\left(\omega - \dot{\theta}_d\right) + \ddot{\theta}_a - ks \tag{8}$$

leads to:

$$\dot{s} = -ks \tag{9}$$

Therefore, the derivative of the Lyapunov function becomes:

$$\dot{V}_3 = -ks^2 \tag{10}$$

This shows that $\dot{V}_3$ is negative definite as long as $k > 0$, ensuring that the Lyapunov function is always decreasing except at the equilibrium $s = 0$, indicating global asymptotic stability.

By utilizing this Lyapunov function, we demonstrate that the sliding mode control law forces the system towards the sliding surface and maintains stability, with the rate of convergence to the desired trajectory θ_d being adjustable via the parameter k.

We hope this explanation clarifies the rationale behind our choice and the definition of the Lyapunov function in our control system design. Thank you for allowing us to enhance the manuscript with this detailed explanation.

16. **Comment 16:** *"In line 183 - 185 the desired reference should be in super_subscript, it shows you missed the underline in latex"*

Response:

Thank you for bringing the formatting issue in lines 183-185 of our manuscript to our attention. We acknowledge the oversight regarding the incorrect representation of the desired reference, which should have been formatted with subscripts and superscripts in LaTeX.

We have corrected this error by properly formatting the LaTeX code to ensure that the reference appears as intended with the correct super_subscript notation. This change will enhance the clarity and accuracy of the mathematical expressions in our manuscript.

Modification

......

Design of tracking control algorithm

......

$$\begin{cases} \dot{z}_1 = z_2 \\ \dot{z}_2 = v_1 \\ \dot{w}_1 = w_2 \\ \dot{w}_2 = v_2 \end{cases} \tag{11}$$

Control laws are designed for the z and w subsystems in equation (11). First, for the z subsystem, let the reference value of z_1 be z_{1d}, and define the errors as $\tilde{z}_1 = z_1 - z_{1d}$ and $\dot{\tilde{z}}_1 = z_2 - \dot{z}_{1d}$. The model becomes:

We appreciate your meticulous attention to detail, which helps improve the presentation and readability of our work.

17. **Comment 17:** *" In line 186 and line 190, you means Lemma 1 not theorem 1, because there is no theorem you defined."*

Response:
Thank you for your careful reading and pointing out the incorrect references to "theorem 1" in lines 186 and 190 of our manuscript. Upon reviewing these sections, we realize that the correct term should indeed be "Lemma 1," as no theorem is defined in our document.

We have corrected this mistake by replacing "theorem 1" with "Lemma 1" in the specified lines. This correction ensures that the references align accurately with the content presented and maintain the logical consistency of our mathematical framework.

Modification

......

Design of tracking control algorithm

......

According to Lemma 1, if the control law is designed as:

$$v_1 = -\alpha_1 \tanh(k_1\tilde{z}_1 + l_1\tilde{z}_2) - \beta_1 \tanh(l_1\tilde{z}_2) + \ddot{z}_{1d} \tag{12}$$

then $\tilde{z}_1 \to 0$ and $\dot{\tilde{z}}_1 \to 0$ can be achieved, where $\alpha_1, \beta_1, k_1, l_1 > 0$.

Similarly, for the w subsystem, let the reference value of w_1 be w_{1d}, and define the errors as $\tilde{w}_1 = w_1 - w_{1d}$ and $\dot{\tilde{w}}_1 = w_2 - \dot{w}_{1d}$. The model becomes:

$$\begin{cases} \dot{\tilde{w}}_1 = \tilde{w}_2 \\ \dot{\tilde{w}}_2 = v_2 - \ddot{w}_{1d} \end{cases} \tag{13}$$

Again, according to Lemma 1, if the control law is designed as:

$$v_2 = -\alpha_2 \tanh(k_2\tilde{w}_1 + l_2\tilde{w}_2) - \beta_2 \tanh(l_2\tilde{w}_2) + \ddot{w}_{1d} \tag{14}$$

We appreciate your attention to detail, which has significantly contributed to the precision and clarity of our manuscript.

18. **Comment 18:** *" Please define one Lemma about what is Lipschitz, and how eq. 15 and eq. 17 ensured globally Lipschitz continuous"*

Response:

Thank you for your request to elaborate on the concept of Lipschitz continuity and its application in our control laws as described by equations (15) and (17). We appreciate the opportunity to clarify this important aspect of our analysis.

Definition and Lemma: Lipschitz continuity, a fundamental property in mathematical analysis and control theory, involves a condition where there exists a constant L such that for any two points x and y in a domain, the inequality:

$$|f(x) - f(y)| \leq L|x - y| \tag{15}$$

holds, where L is known as the Lipschitz constant. This property is crucial for ensuring that small changes in the input of a function lead to proportionally small changes in the output, which is vital for the stability and robustness of control systems.

Lemma: If a function $f(x)$ is differentiable and its derivative is bounded, then f is Lipschitz continuous.

This lemma underpins the design of our control laws, which utilize the hyperbolic tangent function, known to be Lipschitz continuous because its derivative, the sech function squared, is bounded between 0 and 1 .

Application to Equations (15) and (17): In our control system design, we use the hyperbolic tangent function in the control laws given by equations (15) and (17) to exploit this Lipschitz property. The control inputs are designed as:

$$v_1 = -\alpha_1 \tanh(k_1\tilde{z}_1 + l_1\tilde{z}_2) - \beta_1 \tanh(l_1\tilde{z}_2) + \ddot{z}_{1d} \tag{16}$$

$$v_2 = -\alpha_2 \tanh(k_2\tilde{w}_1 + l_2\tilde{w}_2) - \beta_2 \tanh(l_2\tilde{w}_2) + \ddot{w}_{1d} \tag{17}$$

Here, the terms involving the hyperbolic tangent functions ensure that the control laws themselves are Lipschitz continuous, thus providing robust stability and reducing sensitivity to small disturbances or model uncertainties. The bounded nature of the hyperbolic tangent function ensures that the control actions do not exhibit large variations with small changes in error states, thereby maintaining system stability and predictability.

By incorporating Lipschitz continuous functions into the control law, we ensure that the feedback system behaves well under a variety of conditions, achieving the desired stability as per the global asymptotic stability criteria.

We hope this explanation adequately addresses your query and provides a clear understanding of how Lipschitz continuity is ensured in our control system design through equations (15) and (17). Thank you for allowing us to enhance the manuscript with this detailed explanation.

19. **Comment 19:** *"Please define shortly what is SMC law in line 217"*

Response:

Thank you for your comment requesting a definition of the Sliding Mode Control (SMC) law used in our study, specifically referenced in line 217 of our manuscript.

Response and Revision: Sliding Mode Control (SMC) is an effective control strategy known for its robustness against model uncertainties and external disturbances. It involves the design of a control law that forces the system's state to converge to and subsequently maintain within a predefined sliding surface. Once on this surface, the system's dynamics are governed by the sliding mode, which ideally reduces system order and enhances stability and performance.

In the context of our study, the SMC law is designed to ensure that the state trajectory of the VTOL aircraft reaches and maintains a sliding condition defined by the sliding surface $s = c\eta + \dot{\eta}$, where $\eta = \theta - \theta_d$ is the angle tracking error. The control law is formulated as:

$$u_2 = -c\left(\omega - \dot{\theta}_d\right) + \ddot{\theta}_a - ks \tag{18}$$

Here, $-c\left(\omega - \dot{\theta}_d\right)$ compensates for the rate of change in the error, $\ddot{\theta}_d$ is the anticipated acceleration, and $-ks$ is the term that ensures the system stays within the sliding surface, thereby enforcing robust control action against disturbances.

Manuscript Revision: To address your comment and enhance clarity, we have added the following definition and explanation directly before the specified line:

"Sliding Mode Control (SMC) is a robust control strategy that designs a discontinuous control action to drive and maintain the system state on a predefined sliding surface, where the system exhibits desirable dynamic properties. In this study, the SMC law, defined by the equation $u_2 = -c(\omega - \dot{\theta}_d\big) + \ddot{\theta}_a - ks$, is implemented to robustly drive the VTOL aircraft's angular dynamics towards the desired trajectory, effectively countering uncertainties and external disturbances."

We hope this addition clarifies the concept and implementation of the SMC law in our study, thereby addressing your request for further definition.

Thank you once again for your insightful feedback, which helps enhance the precision and accessibility of our manuscript.

20. **Comment 20:** *"After remark 4, please start line 224 from new line, as In control rule ......"*

Response:

Thank you for your meticulous attention to the layout and formatting of our manuscript. We appreciate your suggestion to improve the readability and structure of the text.

In response to your comment, we have adjusted the manuscript by starting line 224 on a new line as suggested. This change is made just after Remark 4, beginning with "In control rule..." to ensure a clearer separation of ideas and enhance the flow of the document.

We believe this modification improves the presentation and allows readers to follow the argumentation and methodology more easily. Thank you once again for your valuable feedback, which contributes to the overall quality of our work.

21. **Comment 21:** *"How you select the x3_dot and epsilon in eq 26"*

Response:
Thank you for your query regarding our selection of the $\dot{x}_3$ and ε parameters in equation (26) within our manuscript. We appreciate the opportunity to clarify the methodology and reasoning behind these choices, which are crucial for the performance of the third-order integrator chain differentiator used in our control system.

Selection of $\dot{x}_3$: The parameter $\dot{x}_3$ in the differential equation is designed to complete the thirdorder integrator chain, enabling higher order differentiation of the target signal θ_d. The specific form:

$$\dot{x}_3 = -\frac{k_1}{\varepsilon^3}(x_1 - \theta_\mathrm{d}) - \frac{k_2}{\varepsilon^2}x_2 - \frac{k_3}{\varepsilon}x_3 \tag{19}$$

is chosen based on the sliding mode control theory, which suggests that such a structure can provide robustness against disturbances and model uncertainties. The coefficients k_1, k_2, and k_3 are tuned to achieve desired convergence rates and stability margins. They are typically selected through empirical tuning supported by stability analysis to ensure the differentiator robustly tracks the derivatives of θ_d up to the third order.

Choice of ε **:** The parameter ε plays a critical role in balancing the trade-off between the speed of response and the sensitivity to noise. In the specific setting of $\varepsilon = \frac{1}{100}\left(1 - \mathrm{e}^{-2t}\right)$ for $0 \leqslant t \leqslant 1.0$, we aim to gradually increase the gain of the differentiator as the system progresses from the initial state. This choice helps mitigate the high sensitivity to initial conditions and potential high-frequency noise in the derivative estimates. The functional form of ε is designed to increase non-linearly, which effectively manages the "peaking" phenomenon often observed in high-gain differentiators at startup, thereby smoothing the initial transient response without significantly affecting the dynamic tracking capability.

This approach ensures that the differentiator provides accurate estimations of $\theta_\mathrm{d}, \dot{\theta}_\mathrm{d}$, and $\ddot{\theta}_\mathrm{d}$ in a controlled manner, thereby supporting the precise and robust operation of our control strategy. We hope this explanation addresses your question comprehensively and clarifies the thoughtful considerations behind the selection of $\dot{x}_3$ and ε in our control system design.

Thank you once again for your insightful query, which allows us to better elucidate the depth of our methodology.

22. **Comment 22:** *"In line 239, the equation number should be 15 and 17, please correct it."*

Response:
Thank you for pointing out the incorrect equation references in line 239 of our manuscript. We appreciate your careful reading and attention to detail.

We have reviewed the manuscript and corrected the error. The references previously indicated as incorrect have now been updated to correctly point to equations (15) and (17), ensuring that all related discussions are accurately connected to the appropriate equations.

We are grateful for your help in improving the accuracy and quality of our paper.

23. **Comment 23:** *"It seems that the figures are stretched, please use the proper and default size of figures according to the requirements of the journal."*

    Response:

    Thank you for bringing to our attention the issue with the formatting of the figures in our manuscript. We appreciate your observation regarding the stretching of figures, which could potentially affect the clarity and interpretability of the data presented.

    We have reviewed the figure formatting guidelines provided by the journal and have adjusted all figures to meet the specified requirements. We have ensured that each figure is now presented in its proper and default size, without any distortion, to maintain the integrity and professional appearance of the graphical data.

    These corrections will ensure that the figures accurately reflect our findings and are consistent with the journal's standards for publication. We are grateful for your guidance, which has helped enhance the presentation quality of our manuscript.

24. **Comment 24:** *"Please adjust all the simulation figures in one or two pages, your one figure i.e., Fig 8 in one page, please adjust the figures according to the journal requirements."*

    Response:
    Thank you for your suggestion to consolidate the simulation figures into one or two pages, and for pointing out the specific issue with Figure 8 being on a separate page. We understand the importance of presenting our figures in a clear and organized manner that complies with the journal's formatting requirements.

    In response to your comments, we have reorganized all simulation figures, including Figure 8, to fit within one or two pages as suggested. This adjustment ensures that related figures are grouped together, enhancing the readability and coherence of the visual data. We have made sure that this reorganization adheres to the journal's layout guidelines.

    We appreciate your feedback, which has helped us improve the presentation of our manuscript. Thank you once again for your attention to detail and constructive suggestions.

25. **Comment 25:** *" Need comparison with other state-of-the-art control strategies in your simulation results."*

    Response:
    Thank you for your valuable suggestion to include comparisons with other state-of-the-art control strategies in our simulation results. We appreciate the importance of such comparisons in demonstrating the efficacy and positioning of our proposed methodology within the current research landscape.

    Our current study focused primarily on developing and validating the specific aspects of our control strategy, which is designed to address unique challenges in VTOL aircraft systems. Due to the scope and time constraints of this initial research, we concentrated on demonstrating the feasibility and effectiveness of our approach rather than conducting extensive comparative analyses.

    However, recognizing the significance of your suggestion, we plan to incorporate comprehensive comparative studies in our future work. This will include detailed simulations

and analyses comparing our developed control strategy against other leading methods to clearly highlight the advantages and potential limitations of our approach in a broader context.

We believe that this planned future work will greatly enrich the understanding of our control strategy's performance and contribute further to the field by situating our results within a comparative framework of existing technologies.

Thank you once again for your constructive feedback, which has provided a clear direction for our subsequent research endeavors.

26. **Comment 26:** *"Your simulation code is not working, I tested on my system with MATLAB version 2024a."*

    Response:
    Thank you for your review and for taking the time to test my simulation code. I sincerely apologize for the issues you encountered with MATLAB version 2024a.

    Please note that my simulation code was developed and tested in MATLAB version 2022a, which may be the cause of the incompatibility issues. To address this, I have repackaged the organized code and detailed running instructions, and uploaded them as supplementary material in S1.pdf. I recommend running these codes using MATLAB 2022a to ensure optimal compatibility and performance.

    Additionally, I will promptly test the code in MATLAB 2024a and make adjustments for any potential compatibility issues. Once this is complete, I will update the code and submit the supplementary material again.

    Thank you again for your valuable comments and patience in testing. Please let me know if there are any further issues or additional information required.

    I look forward to your response.

27. **Comment 27:** *"In you didn't try it on hardware than please try the 3D simulation in MATLAB."*

    Response:

    Thank you for your valuable suggestion. We take the recommendation to conduct 3D simulation tests on hardware very seriously and plan to explore this in our future work.

    Currently, our research focuses on developing and validating algorithms in a 2D simulation environment. However, to fully validate our approach and enhance the practicality of our research, we recognize the importance of conducting 3D simulations, which we have prioritized as a key task for our next steps.

    We will implement 3D simulations within the MATLAB environment and test their performance on hardware that more closely resembles real-world application settings in the next phase of our research. This will help us better understand the behavior of our models in three-dimensional space and make necessary adjustments and optimizations to our algorithms.

Thank you for your guidance and advice. We look forward to providing more comprehensive results in our future studies.

We eagerly await further guidance from you.

28. **Comment 28:** *"While the paper addresses stability, there is limited discussion on the robustness of the control system to various disturbances or uncertainties that might be encountered in real-world operations."*

    Response:
    Thank you for your insightful comments regarding the discussion of the robustness of our control system in the manuscript. We appreciate your pointing out the need for a more comprehensive evaluation of how the system performs under various disturbances and uncertainties, which are indeed critical factors in real-world operations.

    In response to your comments, we recognize that our initial discussion did not sufficiently address the robustness of our control strategy against external disturbances and model uncertainties. To remedy this, we have now expanded our discussion to include:

    Robustness Analysis: We have added a new section that details the robustness of our control strategy. This includes theoretical analysis and simulation results that demonstrate how our control system maintains stability and performance in the presence of various types of disturbances and uncertainties. We discuss specific scenarios that might occur in real-world operations, such as sensor noise, aerodynamic disturbances, and payload variations, and explain how our control system mitigates these factors.

    Sensitivity Analysis: We have also included a sensitivity analysis to quantify the system's response to changes in parameters and environmental conditions. This analysis helps to highlight the thresholds within which the control system can operate effectively without degradation of performance.

    We hope that these additions will address your concerns about the robustness of our control system and provide a clearer understanding of its effectiveness in real-world conditions. We are grateful for your feedback, which has undoubtedly enhanced the depth and quality of our manuscript.

    Thank you once again for your valuable suggestions.

29. **Comment 29:** *"The proposed sliding mode control strategy, while theoretically sound, appears to be quite complex. This complexity might pose challenges in real-world implementation and tuning of the control system. How you justify this question?"*

    Response:

    Thank you for your insightful observation regarding the complexity of the proposed sliding mode control (SMC) strategy and its potential implications for real-world implementation and tuning.

    We recognize that the theoretical robustness of our SMC strategy comes with a certain level of complexity, primarily due to the need to effectively handle nonlinearities and uncertainties inherent in VTOL aircraft dynamics. This complexity is essential to achieve

the desired robustness and performance in diverse operational conditions, which simpler control strategies might not adequately address.

Justification and Implementation Strategies:

Necessity of Complexity: The complexity of the control strategy is justified by its ability to ensure high precision and stability in environments where simpler models fail to perform adequately. The design incorporates features necessary to manage the high degrees of freedom and dynamic disturbances typical of VTOL operations, ensuring safety and reliability.

Design for Real-World Application: We have designed the control strategy not only for theoretical robustness but also with practical implementation in mind. This includes modular components that can be independently tuned and validated, facilitating easier adaptation to different types of VTOL aircraft and operational scenarios.

Tuning and Implementation Support: To address potential challenges in tuning and implementation, we provide detailed guidelines on the parameter tuning process, supported by extensive simulation data that illustrate how different settings affect system behavior. These guidelines are designed to help practitioners adjust the control parameters effectively to meet specific operational requirements.

Simplification Through Automation: We are exploring automated tuning methods that use machine learning techniques to adapt the control parameters in real-time based on observed performance. This approach aims to reduce the manual effort required in tuning and maintaining the control system, thus making it more accessible for practical applications.

Training and Support: Recognizing the steep learning curve that might be associated with implementing such a complex system, we propose to develop comprehensive training programs and simulation tools. These resources will assist system operators and engineers in understanding, implementing, and maintaining the control strategy effectively.

We believe that these measures will significantly mitigate the challenges associated with the complexity of the SMC strategy, making it viable and beneficial for real-world applications. We are committed to continuous improvement of the implementation process to ensure that it can be as user-friendly as possible while maintaining the integrity and capabilities of the control system.

Thank you again for your constructive critique, which prompts us to clarify and enhance the applicability of our research.

30. **Comment 30:** *" Please add one Figure, in which it will show the schematic diagram, in which show the flow of control to actuators of VTOL, remove the Fig. 1 and add one complete schematic diagram in which you put the vtol in the place of system or plant."*

Response:

Thank you for your valuable feedback on our manuscript. As per your suggestion, we have removed Figure 1 from the manuscript. To address the request for a schematic diagram illustrating the flow of control to the actuators in a VTOL system, we have prepared a new figure. This figure has been added to the supplementary materials accompanying our revised submission.

We believe that this new diagram effectively illustrates the control mechanisms as you requested, providing a clearer understanding of our system's operation.

We appreciate your guidance in improving our manuscript and hope that these revisions meet your expectations.

31. **Comment 31:** *" English is very weak, Need to much improvements."*

    Response:
    Thank you for your feedback and for pointing out the concerns regarding the English language quality in our manuscript. We understand the importance of clear and accurate communication, especially in scientific publications.

    To address this issue, we have carefully revised the manuscript for language clarity and correctness. Additionally, we have enlisted the assistance of a professional English language editing service to ensure that the text meets the high standards required for publication.

    We appreciate your attention to detail and your help in improving our manuscript. We believe these revisions will greatly enhance the readability and overall quality of our paper.

    Thank you once again for your valuable comments.

32. **Comment 32:** *" Grammer and punctuations also need to be improved."*

    Response:
    Thank you for your careful review and for highlighting the grammar and punctuation issues in our manuscript. We apologize for the oversights and recognize the importance of these elements in ensuring the manuscript's clarity and professionalism.

    To address your concerns, we have thoroughly revised the manuscript to correct the grammatical and punctuation errors. Additionally, we have engaged the services of a professional editor who specializes in academic publications to ensure that our text meets the high standards of scholarly communication.

    We are grateful for your guidance that helps enhance the quality of our paper, and we believe that these revisions have significantly improved the readability and accuracy of our manuscript.

    Thank you again for your constructive comments.

    The authors sincerely appreciate the reviewer's detailed and insightful feedback, which has significantly contributed to improving both the quality and clarity of our manuscript. We are truly grateful for the time and effort invested in offering constructive criticism and valuable suggestions, helping us enhance both the substance and presentation of our work.

References

[1] I. U. Haq, Q. Khan, S. Ullah, S. A. Khan, R. Akmeliawati, M. A. Khan, and J. Iqbal, "Neural network-based adaptive global sliding mode mppt controller design for stand-alone photovoltaic systems," *Plos one*, vol. 17, no. 1, p. e0260480, 2022.

[2] S. Irfan, L. Zhao, S. Ullah, U. Javaid, and J. Iqbal, "Differentiator-and observer-based feedback linearized advanced nonlinear control strategies for an unmanned aerial vehicle system," *Drones*, vol. 8, no. 10, p. 527, 2024.

[3] M. Kuprikov and L. N. Rabinskiy, “Vertical take-off and landing aircrafts: Myth or reality of modern aviation,” *Journal of Mechanical Engineering Research and Developments*, vol. 41, no. 4, pp. 46–52, 2018.

[4] A. Intwala and Y. Parikh, “A review on vertical take off and landing (vtol) vehicles,” *International Journal of Innovative Research in Advanced Engineering (IJIRAE)*, vol. 2, no. 2, pp. 187–191, 2015.

[5] A. Misra, S. Jayachandran, S. Kenche, A. Katoch, A. Suresh, E. Gundabattini, S. K. Selvaraj, and A. A. Legesse, “A review on vertical take-off and landing (vtol) tilt-rotor and tilt wing unmanned aerial vehicles (uavs),” *Journal of Engineering*, vol. 2022, no. 1, p. 1803638, 2022.

[6] H. Gu, X. Lyu, Z. Li, S. Shen, and F. Zhang, “Development and experimental verification of a hybrid vertical take-off and landing (vtol) unmanned aerial vehicle (uav),” in *2017 International Conference on Unmanned Aircraft Systems (ICUAS)*. IEEE, 2017, pp. 160–169.

[7] A. Bacchini and E. Cestino, “Key aspects of electric vertical take-off and landing conceptual design,” *Proceedings of the Institution of Mechanical Engineers, Part G: Journal of Aerospace Engineering*, vol. 234, no. 3, pp. 774–787, 2020.

[8] D. Orbea, J. Moposita, W. G. Aguilar, M. Paredes, R. P. Reyes, and L. Montoya, “Vertical take off and landing with fixed rotor,” in *2017 CHILEAN Conference on Electrical, Electronics Engineering, Information and Communication Technologies (CHILECON)*. IEEE, 2017, pp. 1–6.

[9] R. Oung and R. D’Andrea, “The distributed flight array: Design, implementation, and analysis of a modular vertical take-off and landing vehicle,” *The International Journal of Robotics Research*, vol. 33, no. 3, pp. 375–400, 2014.

[10] F. Çakici and M. K. Leblebicioğlu, “Control system design of a vertical take-off and landing fixed-wing uav,” *IFAC-papersonline*, vol. 49, no. 3, pp. 267–272, 2016.

[11] M. I. Ullah, S. A. Ajwad, M. Irfan, and J. Iqbal, “Non-linear control law for articulated serial manipulators: Simulation augmented with hardware implementation,” *Elektronika ir Elektrotechnika*, vol. 22, no. 1, pp. 3–7, 2016.

[12] R. O. Saber and R. M. Murray, “Flocking with obstacle avoidance: Cooperation with limited communication in mobile networks,” in *42nd IEEE International Conference on Decision and Control (IEEE Cat. No. 03CH37475)*, vol. 2. IEEE, 2003, pp. 2022–2028.

[13] R. Olfati-Saber, J. A. Fax, and R. M. Murray, “Consensus and cooperation in networked multi-agent systems,” *Proceedings of the IEEE*, vol. 95, no. 1, pp. 215–233, 2007.

[14] F. Bullo and K. M. Lynch, “Kinematic controllability for decoupled trajectory planning in underactuated mechanical systems,” *IEEE Transactions on Robotics and Automation*, vol. 17, no. 4, pp. 402–412, 2001.

[15] F. Nafa, S. Labiod, and H. Chekireb, “Direct adaptive fuzzy sliding mode decoupling control for a class of underactuated mechanical systems,” *Turkish Journal of Electrical Engineering and Computer Sciences*, vol. 21, no. 6, pp. 1615–1630, 2013.

[16] B. Ata and R. Coban, “Decoupled adaptive backstepping sliding mode control of underactuated mechanical systems,” *Journal of Control Engineering and Applied Informatics*, vol. 24, no. 1, pp. 45–56, 2022.

[17] P. Pustina, C. Della Santina, and A. De Luca, “Feedback regulation of elastically decoupled underactuated soft robots,” *IEEE Robotics and Automation Letters*, vol. 7, no. 2, pp. 4512–4519, 2022.

[18] G. Sonugür, “A review of quadrotor uav: Control and slam methodologies ranging from conventional to innovative approaches,” *Robotics and Autonomous Systems*, vol. 161, p. 104342, 2023.

[19] A. S. Hanif, X. Han, and S.-H. Yu, “Independent control spraying system for uav-based precise variable sprayer: A review,” *Drones*, vol. 6, no. 12, p. 383, 2022.

[20] W. J. Yun, S. Park, J. Kim, M. Shin, S. Jung, D. A. Mohaisen, and J.-H. Kim, “Cooperative multiagent deep reinforcement learning for reliable surveillance via autonomous multi-uav control,” *IEEE Transactions on Industrial Informatics*, vol. 18, no. 10, pp. 7086–7096, 2022.

[21] B. Ma, Z. Liu, Q. Dang, W. Zhao, J. Wang, Y. Cheng, and Z. Yuan, “Deep reinforcement learning of uav tracking control under wind disturbances environments,” *IEEE Transactions on Instrumentation and Measurement*, vol. 72, pp. 1–13, 2023.

[22] G. J. Ducard and M. Allenspach, “Review of designs and flight control techniques of hybrid and convertible vtol uavs,” *Aerospace Science and Technology*, vol. 118, p. 107035, 2021.

[23] X. Wang, J. Liu, and K.-Y. Cai, “Tracking control for a velocity-sensorless vtol aircraft with delayed outputs,” *Automatica*, vol. 45, no. 12, pp. 2876–2882, 2009.

[24] R. Olfati-Saber, “Global configuration stabilization for the vtol aircraft with strong input coupling,” *IEEE transactions on Automatic Control*, vol. 47, no. 11, pp. 1949–1952, 2002.
