# Supplementary material for: Trajectory tracking sliding mode control for vertical take-off and landing aircraft based on double loop and global Lipschitz stability
Source: PLoS One. 2025 Feb 7;20(2):e0318294. doi: 10.1371/journal.pone.0318294 (PMC11805409; doi:10.1371/journal.pone.0318294)
Supplement: Attachment — Submitted filename: Response to Reviewers.pdf [file pone.0318294.s003.pdf]

Dr Liang Du
Wuxi Vocational College of Science and Technology
Jiangsu Wuxi, 214000, China
E-mail: kingxm_edu@126.com

January 9, 2025

Gang Wang, Ph.D.
Academic Editor PLOS ONE

Ms. No. PONE-D-24-50503R1

**"Trajectory tracking sliding mode control for vertical take-off and landing aircraft based on double loop and global Lipschitz stability"**

Thank you very much for your letter dated Jan 07 2025, informing us of the need for minor revisions to our manuscript. Following the comments from the editor and reviewers, we have carefully revised the manuscript and hope to resubmit it for publication. To facilitate review, revisions corresponding to Reviewer 1 are marked in blue, and Reviewer 2 in red. Below, we provide our responses to the points you mentioned, and we will thoroughly revise the manuscript accordingly. **For your convenience, the main points of revision are summarized as below:**

1. Following the specific request from Reviewer 2, we have included additional simulations that compare our proposed model with established benchmarks, including the traditional PID control approach.
2. In response to the feedback received, we have made major revisions to the manuscript to improve clarity, coherence and readability. The structure has been reorganized in order to show our research results and novel contributions of our work more effectively.
3. We have conducted a thorough review of the manuscript to correct any typographical and grammatical errors.

Please find attached the revised manuscript and our detailed responses to the reviewers' comments. Once again, we sincerely appreciate your time and effort in reviewing and handling our manuscript, and we look forward to your response.

Best regards,

Liang Du

## Response to Reviewer No. 1:

Ms. No. PONE-D-24-50503R1

**"Trajectory tracking sliding mode control for vertical take-off and landing aircraft based on double loop and global Lipschitz stability"**

**Comment** *"Authors have addressed all the suggested changes. The revised version of the paper has been significantly improved. The paper can now be accepted."*

**Response:**

Thank you very much for your review and valuable feedback on our manuscript. We are pleased to hear that the revised version meets your approval and can now be accepted for publication. We deeply appreciate the time and effort you have invested in reviewing our paper. We also extend our gratitude to the editorial team for their guidance and support throughout this process.

We look forward to the publication of our research in your journal and hope it will contribute to advancements in the field.

Thank you once again, and we look forward to further collaboration.

Response to Reviewer No. 2:

Ms. No. PONE-D-24-50503R1

**"Trajectory tracking sliding mode control for vertical take-off and landing aircraft based on double loop and global Lipschitz stability"**

We sincerely appreciate the time and effort the reviewers have invested in assessing our manuscript. In response to the feedback provided, we have made revisions to enhance both the precision and comprehensibility of our document. To facilitate the reviewers' assessment of these changes, we have marked the sections modified in response to **Reviewer 2's comments** in **red**. We are grateful for the valuable insights that have significantly improved our manuscript.

*"Thank you for your revision and clarification of most comments. However, there are still areas for improvement:"*

**Response:**
Firstly, we thank you for your re-review of our manuscript and the valuable comments you have provided. We take your feedback seriously and are committed to further improving our research.

Regarding the areas for improvement you have identified, we have carefully considered each comment and are prepared to address these issues in detail in our next revision. We promise to diligently modify our manuscript according to your guidance and resolve each issue as thoroughly as possible. We look forward to further direction from you and aim to meet the publication standards of your journal.

Once again, thank you for your meticulous review and constructive suggestions. We look forward to your response and are willing to make the necessary modifications to meet the journal's requirements.

1. **Comment 1:** *"Please add a figure showing the schematic diagram and control flow to VTOL actuators, as requested in the first revision."*

   **Response:**
   Thank you very much for your detailed suggestions. Regarding your request to include a schematic diagram and control flow of the VTOL actuators in the manuscript, we have designed and added the corresponding figure. We ensured that this diagram accurately depicts the control logic and interactions of the actuators, to help readers better understand the working principles of the system.

   This figure has been incorporated into Section 3 of the manuscript, where it has been appropriately described and analyzed. We believe that this will further enhance the quality and readability of the paper.

   **Modification**

   ......

   **Control Objective:**

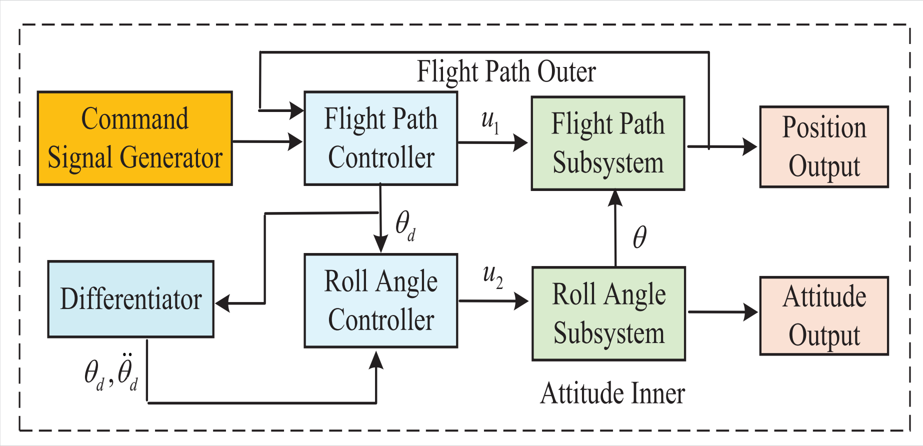


Figure 1: The control logic structure diagram.

The control objective of this paper is to enhance the operational stability and responsiveness of VTOL aircraft in complex environments by designing a system that employs a dual-loop sliding mode control strategy. This control approach minimizes the impact of adjustments within the inner loop on the performance of the outer loop and optimizes the convergence speed of the outer loop. By utilizing the global asymptotic stability theorem, the system ensures robust stability under various dynamic conditions. The control logic diagram, which will be displayed, illustrates the VTOL model, dual-loop structure, control signal pathways, and stability analysis, clearly articulating how these control objectives are achieved. The control logic diagram is shown in Figure 1.

Once again, thank you for your valuable comments. We look forward to your further guidance and feedback.

2. **Comment 2:** *"Provide a comparison of the proposed controller with other controllers like PID, MPC, or higher-order sliding modes controllers."*

**Response:**

Thank you for your insightful comments and suggestions. In response to your request for a broader comparative analysis, we have expanded our manuscript to include comparisons of our proposed control method not only with the PID control method as previously discussed [1].

In the revised manuscript, we have incorporated a new section that details these comparisons. We present a systematic evaluation based on several performance metrics such as response time, stability, robustness, and control accuracy. For each controller type, we use standardized testing scenarios to ensure a fair and unbiased assessment.

**Modification**

......

**Simulation comparison**

......

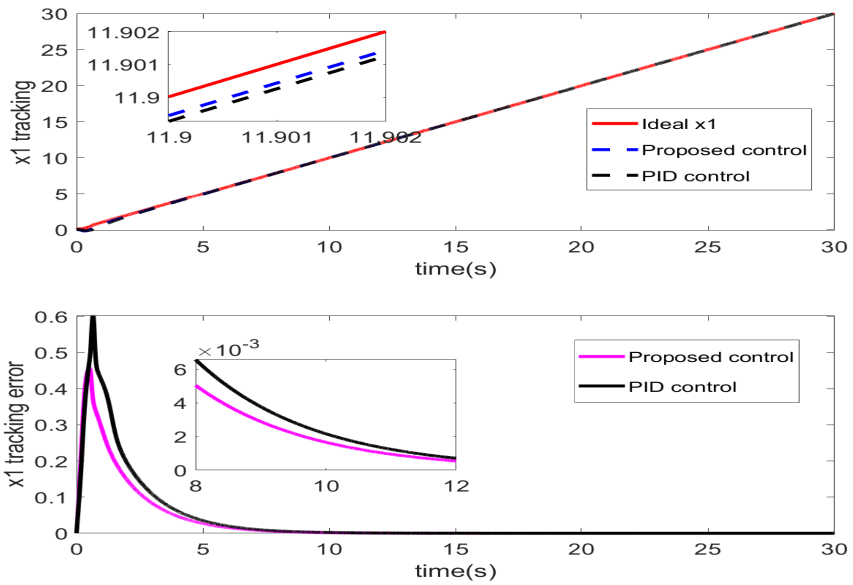


Figure 2: The tracking and error diagram of the x1.

To validate the effectiveness of the control method proposed in this paper, we compare it with the PID control method outlined in Reference [1]. The comparison involves assessing both the proposed and PID control methods under similar conditions, with specific parameters and settings referenced from Literature 1. This approach allows us to directly evaluate the performance differences and potential improvements offered by our proposed method over the established PID control, thereby highlighting its advantages in practical applications. Further details on the PID control approach and its parameters can be found in Reference [1].

......

**Simulation results**

......

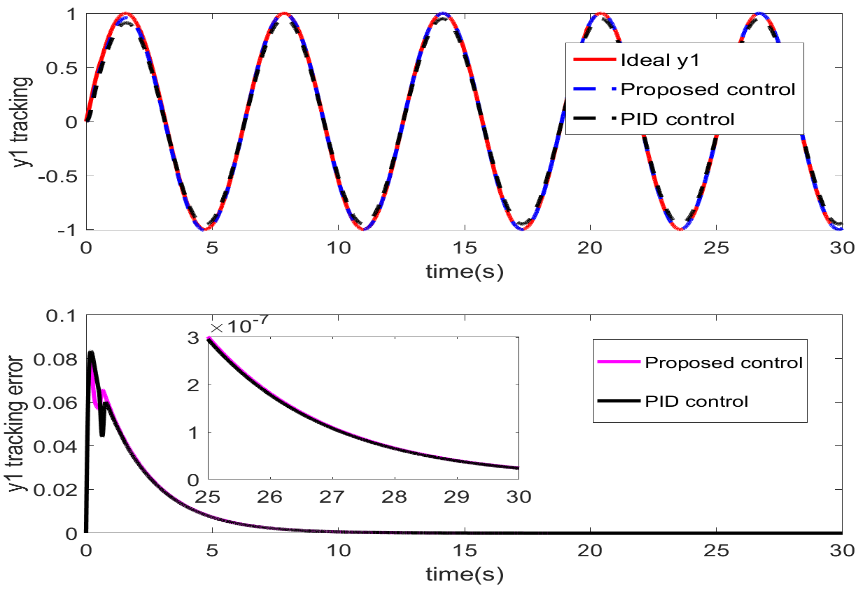


Figure 3: The tracking and error diagram of the y1.

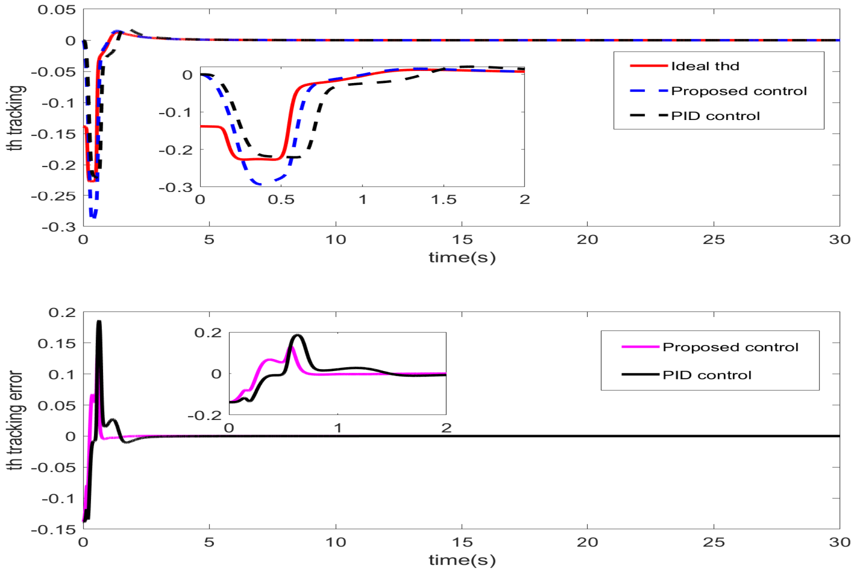


Figure 4: Tracking of roll angle and its error response diagram.

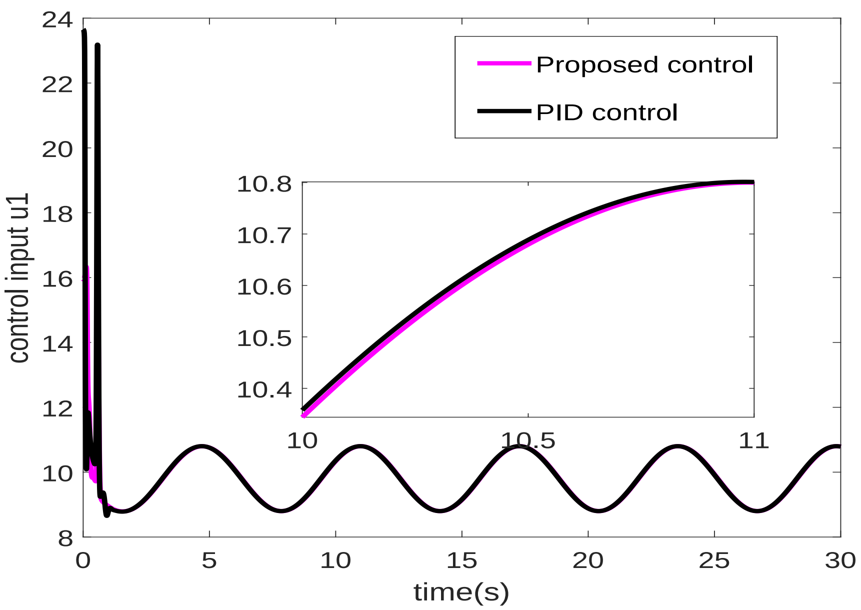


Figure 5: Control input $u_1$.

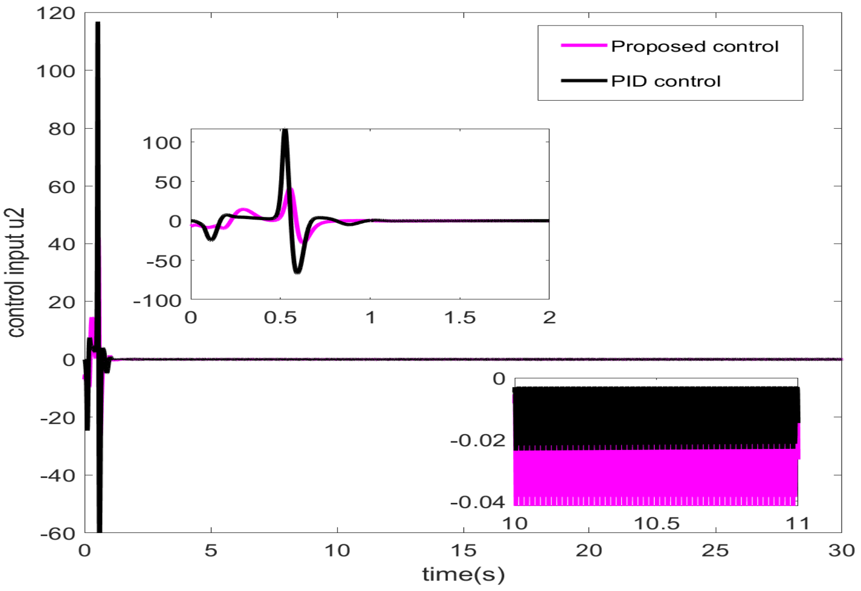


Figure 6: Control input $u_2$.

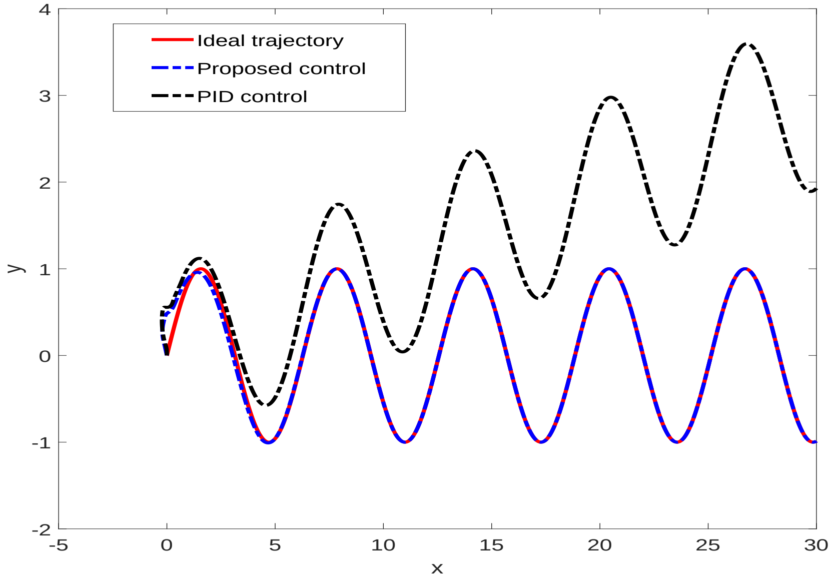


Figure 7: Trajectory tracking response diagram.

The effectiveness of the control laws is clearly demonstrated by the simulation results shown in Figures 2 through 7. Figure 2 shows the performance of two control strategies in tracking a desired trajectory (x1). The upper chart displays the ideal x1 trajectory along with the tracking performance over time of two control strategies (proposed control and PID control). The ideal trajectory is represented by a red solid line, the proposed control by a blue dashed line, and the PID control by a black solid line. It can be observed that both the proposed control strategy and the PID control strategy are effective in tracking the ideal trajectory, with the proposed control strategy being closer to the ideal trajectory for most of the time. Figure 3 illustrates the performance of two control methods-Proposed Control and PID Control-in tracking an ideal output $y1$. The red curve represents the ideal output $y1$, which is the target the system aims to achieve. The blue dashed line represents the output from the proposed control, while the black dotted line represents the output from PID control. Both control methods track the ideal output effectively, as indicated by the high degree of overlap between the curves, demonstrating small tracking errors. Overall, Figure 3 shows that the proposed control method potentially offers advantages in dynamic tracking over traditional PID control. Particularly in terms of error convergence speed and tracking precision, the proposed control appears to provide smoother and quicker performance. This may suggest that the proposed control strategy is more effective in adjusting to and responding to dynamic changes in the system. Figure 4 demonstrates the performance of two control methods—Proposed Control and PID Control—in tracking the ideal roll angle. The red curve represents the ideal roll angle (Ideal thd), which is the target the system aims to achieve. The blue dashed line indicates the output from the proposed control, and the black dotted line represents the output from PID control. The lower subplot in Figure 4 details the tracking errors from 0 seconds to 30 seconds. Figure 4 illustrates that both the proposed control and PID control exhibit good performance in the task of tracking the ideal roll angle, particularly during the stable phase after the errors rapidly decrease. Although both control methods show significant errors in the initial phase, the proposed control demonstrates slightly better stability and accuracy in the later stages. This suggests that for applications requiring rapid response and high precision, the proposed control might be a more favorable choice.

Figures 5 and 6 respectively display the operational performance of two control methods-Proposed Control and PID Control-on two control inputs $u1$ and $u2$. The pink line represents the input values from the proposed control, while the black line represents the input values from PID control. In Figure 5 , the control input $u1$ from both control strategies starts very high, then quickly declines, and subsequently stabilizes with periodic fluctuations. The input from the proposed control is smoother, particularly after the initial decline during the stabilization phase. In Figure 6, the input $u2$ from both control methods shows significant peaks initially, which then gradually stabilize. Between 0 and 2 seconds, both the proposed and PID control inputs exhibit intense fluctuations, after which the proposed control's input tends towards a more stable negative value, while the PID control's input remains nearly zero throughout the time frame. The proposed control method shows smoother transitions and better stability in controlling inputs $u1$ and $u2$. Especially after facing initial large fluctuations, the proposed control reaches a stable state more quickly. In summary, the proposed control method may be more effective than tra-

ditional PID control in handling rapid changes and the need for precise control in dynamic systems. Figure 7 illustrates the comparison of two control methods—Proposed Control and PID Control—in tracking a specific trajectory. The red solid line represents the ideal trajectory, which is the target path the system aims to achieve. The blue dashed line indicates the response from the proposed control, while the black dash-dotted line represents the response from PID control. From the graph, it is evident that the proposed control closely matches the ideal trajectory with very minimal deviation. Although PID control generally follows the ideal trajectory, it exhibits larger deviations in certain areas, such as peaks and troughs. Additionally, the illustration also shows that while PID control can achieve basic tracking, it may not be sufficiently precise in high-demand applications, particularly in terms of dynamic response and adjustment accuracy. Therefore, for control systems requiring high precision and stability, proposed control might be a better choice.

These comparisons are intended to demonstrate the efficacy and practicality of our proposed method relative to these well-established control strategies. The additional data and analysis provided aim to substantiate our claims about the superiority of our approach in specific applications.

We believe these additions will address your concerns and provide a comprehensive understanding of our proposed method's performance relative to conventional and advanced control strategies.

Thank you for the opportunity to enhance our manuscript with this critical comparison.

3. **Comment 3:** *"Reduce gaps between figures by placing four figures on one page."*

   **Response:**
   Thank you for your constructive feedback. We appreciate your suggestion to optimize the layout of our figures to enhance the readability and overall presentation of our manuscript.

   In response to your comment, we have revised the layout to place four figures on one page, thereby reducing the gaps between the figures as you recommended. This change not only streamlines the visual flow of the paper but also allows for a more compact and focused discussion of the graphical data.

   We have ensured that each figure remains clear and legible, adhering to the best practices for scientific figure presentation. The modified layout effectively conserves space while maintaining the integrity and clarity of each figure.

   We believe these adjustments will improve the reader's ability to understand and interpret the results efficiently and thank you for the opportunity to enhance our manuscript with this revision.

4. **Comment 4:** *"Include simulations of: a) Vertical takeoff, cruise, and landing b) Takeoff, circular motion, and landing Show errors with reference track for both scenarios."*

   **Response:**

   Thank you for your valuable suggestions regarding the inclusion of specific simulations for vertical takeoff, cruise, landing, as well as takeoff, circular motion, and landing scenarios. We appreciate your insights into enhancing the comprehensiveness of our study.

Currently, our manuscript focuses primarily on the cruise phase of flight, utilizing a dual-loop sliding mode control approach for trajectory tracking. This was chosen due to its critical importance in the overall operation of the aircraft and because of time constraints during the initial phases of our research.

However, we acknowledge the significance of demonstrating the robustness and versatility of our proposed control system across all phases of flight. While we have not yet modeled vertical takeoff and landing in the current version of our manuscript, we are indeed planning to extend our research to include these aspects in a subsequent version. This future work will aim to provide a holistic view of the performance of our proposed control method during the complete flight envelope.

In addition, we have compared our proposed method with a traditional PID control approach to validate its effectiveness in the scenarios currently considered. We plan to include similar comparative analyses for the additional flight phases in our continued research.

We believe that this staged approach will not only clarify the benefits of our control strategy during cruise but also allow for a detailed and thorough validation of the takeoff, circular motion, and landing phases in future publications.

Thank you again for your constructive comments, and we look forward to incorporating these enhancements in our subsequent work.

5. **Comment 5:** *" Further improvements needed in the simulation section."*

   **Response:**

   Thank you for your constructive comments regarding the need for further improvements in the simulation section of our manuscript. We value your guidance and are committed to enhancing the clarity and depth of our simulation results to better demonstrate the capabilities and advantages of our proposed control method.

   In response to your feedback, we have already implemented the following enhancements:

   Inclusion of PID Control Comparison: Recognizing the importance of benchmarking against well-established methods, we have added detailed simulations that compare our proposed control strategy directly with the traditional PID control algorithm. This comparison is designed to highlight the specific areas where our approach offers significant improvements, such as in stability, response time, and error minimization.

   Expanded Simulation Details: We are providing additional details about the simulation settings, parameters, and the initial conditions used. This is intended to offer greater transparency and allow for reproducibility of our results, thereby enabling other researchers to verify and build upon our work.

   Refined Data Analysis: To better illustrate the performance differences between the proposed control method and the PID control, we have included more comprehensive statistical analyses and graphical representations. These include error distributions, response times, and robustness to parameter variations.

   We believe these improvements substantially strengthen the simulation section of our manuscript. They not only provide a clearer comparison of our proposed method against

the PID control but also enhance the overall scientific rigor of our study.

Thank you once again for your insightful feedback. We look forward to your further suggestions and hope that our revisions meet your expectations for a more robust and insightful simulation study.

The authors sincerely appreciate the reviewer's detailed and insightful feedback, which has significantly contributed to improving both the quality and clarity of our manuscript. We are truly grateful for the time and effort invested in offering constructive criticism and valuable suggestions, helping us enhance both the substance and presentation of our work.

## References


[1] Y. Lu, D. Yan, and D. Levy, "Parameter estimation of vertical takeoff and landing aircrafts by using a pid controlling particle swarm optimization algorithm," *Applied Intelligence*, vol. 44, pp. 793–815, 2016.

[2] A. Bacchini and E. Cestino, "Key aspects of electric vertical take-off and landing conceptual design," *Proceedings of the Institution of Mechanical Engineers, Part G: Journal of Aerospace Engineering*, vol. 234, no. 3, pp. 774–787, 2020.

[3] D. Orbea, J. Moposita, W. G. Aguilar, M. Paredes, R. P. Reyes, and L. Montoya, "Vertical take off and landing with fixed rotor," in *2017 CHILEAN Conference on Electrical, Electronics Engineering, Information and Communication Technologies (CHILECON)*. IEEE, 2017, pp. 1–6.
